# Phase response curve and RNA-sequencing demonstrate spiders’ sensitivity to light and pinpoint candidate light-responsive genes

**DOI:** 10.1371/journal.pone.0344063

**Published:** 2026-09-23

**Authors:** Natalia Toporikova, Wenduo Cheng, Leyuan Qian, Andrew Mah, Thomas Clarke, Thomas C. Jones, Darrell Moore, Nadia A. Ayoub

**Affiliations:** 1 Department of Biology, Washington and Lee University, Lexington, Virginia, United States of America; 2 Department of Biological Sciences, East Tennessee State University, Johnson City, Tennessee, United States of America; Karlsruhe Institute of Technology: Karlsruher Institut fur Technologie, GERMANY

## Abstract

Spiders can maintain a wide range of free-running periods while still being entrained to a 24-hour day. To investigate the underlying mechanism of this entrainment, we constructed the phase-response curve (PRC) for the orb weaver, *Metazygia wittfeldae*, by subjecting the spiders to one-hour light pulses at various times throughout the circadian day. The resulting type 0 PRC showed high amplitude (> 6 hour) phase advance and delays when the light pulse was applied during circadian time (CT) 16–18, including a break point of delays to advances. We then investigated the genetic mechanism of response to light by splitting *M. wittfeldae* adult females entrained to 12 hours light:12 hours dark (LD 12:12) into two groups. One group received a 1-hour light pulse 5 hours after lights off (equivalent to CT17), and the other group did not. We then sacrificed spiders for RNA isolations 1 and 10 hours after the light pulse. We identified numerous genes that were downregulated by the light pulse 1 hour after the pulse relative to no pulse group. Intriguingly, many of these genes had a flipped pattern of expression 9 hours later – the pulse group had higher expression than the no pulse group. We also identified clock gene homologs in *M. wittfeldae* that had distinct expression patterns from other arthropods.

## Introduction

Daily rhythms are ubiquitous across life, from the sleep-wake cycle in humans [1] to cell division in fungi [2] to nitrogen fixation in cyanobacteria [3]. These rhythms are termed circadian if they follow a natural day-night environment and continue to cycle for approximately (circa) 24 hours even in constant conditions. Rhythms that continue without environmental stimuli are likely controlled by an endogenous circadian clock, which may provide a fitness advantage by allowing organisms to anticipate and prepare for daily changes in their environment [4]. The proteins that control the endogenous circadian clock differ between plants, fungi, animals, and cyanobacteria, suggesting that clocks may have evolved independently multiple times, further supporting their evolutionary benefit [5].

Despite differences in individual components, all molecular clocks share a common feature: a transcription-translation negative feedback loop [6]. For instance, in the fruit fly, *Drosophila melanogaster*, the clock genes *period* (*per*) and *timeless* (*tim*) form a negative feedback loop by encoding proteins that inhibit their own transcription. Transcription of *per* and *tim* is activated by a heterodimer of basic helix-loop-helix transcription factors, CLOCK (CLK) and CYCLE (CYC), which bind the E-box in the *per* and *tim* promoters [7]. After translation, PER and TIM proteins dimerize, enter the nucleus, and inhibit CLK and CYC from binding the E-boxes. Once PER and TIM degrade, CLK and CYC can again promote transcription [8–12]. This feedback loop generates daily cycles of *per* mRNA and PER protein, which in turn influence the daily cycles of locomotion and other physiological processes [13]. The transcription-translation feedback loop described above is conserved across animals, with some differences: in mammals, butterflies, and many other arthropods, BMAL1 (a homolog of CYC) dimerizes with CLK to promote transcription of *per* and *cryptochrome* genes, termed mammalian-type *cry* or *cry2* in arthropods. PER and CRY2 then dimerize and enter the nucleus to block transcription [14–16]. Arthropods, including *D. melanogaster*, possess a *cryptochrome* paralog, *cry1*, whose protein product (CRY1) functions as a blue-light photoreceptor rather than as a transcriptional repressor [17].

The environment also affects the transcription-translation feedback loop. Since the endogenous circadian period, called the free-running period (FRP), is not exactly 24 hours, external cues must entrain the clock to the earth’s rotational period [18]. These environmental cues, such as daily temperature or light changes, are called zeitgebers (time givers [19]). Light is the best described and probably the most powerful zeitgeber. In mammals, light reaching the eyes changes the structure of melanopsin, which in turn starts a cascade that eventually results in higher transcription of *per* genes [20–25]. In fruit flies, light directly changes the structure of CRY1 so that it binds TIM, leading to the ubiquitination and, thus, degradation of TIM [17]. Without TIM, PER becomes unstable so that it cannot block transcription of CLK and CYC, essentially resetting the clock every morning. The compound eyes also mediate light entrainment in fruit flies, but through a mechanism that is not well understood [26].

The phase response curve (PRC) is a classic method for investigating the nature of the endogenous circadian cycle and its entrainment by light [27,28]. Essentially, the effect of light on the cycle differs depending on the point in the cycle (phase) at which light is applied. At some points of the cycle, light can shift the phase, while at others, it cannot [29,30]. For instance, in mammals, light in the early morning advances the phase by promoting the transcription of *per* genes slightly earlier than it would naturally. On the other hand, light in the evening delays the phase by increasing transcription of *per* genes [31–33]. Light pulses in the middle of the subjective day, even in a constantly dim environment, typically do not shift a cycle in humans [29,34]. Similarly, fruit flies experience phase delays if pulsed with light in the early evening, when *per* and *tim* are rising. In contrast, flies experience phase advances if pulsed with light in the late evening when *per* and *tim* would be naturally decreasing [35,36].

The largest values (or amplitude) of phase advances or delays in a PRC reflect the strength of the circadian oscillator and/or its sensitivity to light [37]. In wild-type flies with intact *cry1*, the PRC has much higher amplitude advances and delays than in *cry1*-mutants [38]. Mutants without functional CRY1 still experience low amplitude phase shifts, presumably due to low-sensitivity compound eye-mediated light detection [26,38]. A high amplitude PRC can also be indicative of a weak oscillator [39]. For instance, clock mutants with low differences between the peak and trough of *per* expression have high amplitude PRCs [40]. Mechanistically, a low-amplitude oscillator can be driven across or beyond its limit cycle by a stimulus of moderate strength, producing the discontinuous, large-amplitude shifts characteristic of a type 0 PRC; conversely, a strong oscillator absorbs such perturbations and yields small, continuous shifts of a type 1 PRC [39,41].

Spiders are an emerging system for investigating the evolution of circadian rhythms and their entrainment. Thus far, circadian rhythmicity of locomotor activity has been well documented in multiple species from 6 divergent families of spiders [42–49]. Anti-predator behavior in one orb-weaving species is also circadian [50], which appears to be driven by circadian cycling in octopamine levels [51]. Most spiders also build their webs on a daily cycle [52], but the persistence of the rhythm in constant conditions has not yet been shown.

Surprisingly, the FRP of locomotor activity can be highly variable among and within spider species. For instance, the trashline orb weaver *Cyclosa turbinata* (Araneidae) has an average FRP of 18.7 hours in constant darkness, the shortest known period for any free-living organism. In addition, the ranges of FRPs exhibited by three cobweb weaving spider species (Theridiidae) were large: 19–23.5 h in the common house spider *Parasteatoda tepidariorum* (mean FRP = 21.7 h), 20–29 h in the subsocial spider *Anelosimus studiosus* (mean FRP = 23.1), and 20–30.1 h (mean FRP = 24.5) in the southern black widow *Latrodectus mactans* [49]. Even the orb weaver *Metazygia wittfeldae* (Araneidae), which possesses an almost “typical” FRP (mean = 22.7 h) varied from 19–24 hours [48]. The three theridiid and two araneid species have FRPs that vary an order of magnitude more than those of most animals examined thus far [49]. Nevertheless, all the individuals of these species rapidly entrained to cycles of 12 hours light, 12 hours dark (LD 12:12) [47–49].

The robust entrainment to light by spiders with divergent FRPs is consistent with a weak oscillator or a heightened sensitivity to light relative to other arthropods. However, very little is known about clock mechanisms in spiders. We thus used a two-pronged approach to investigate the circadian cycle in one orb-weaving species, *M. wittfeldae*. First, we constructed a PRC to determine when the system is most sensitive to light and to discover the level of light response. Second, we used the results of the PRC to determine the optimal time to expose spiders to a light pulse for RNA sequencing. We discovered *M. wittfeldae* has the highest amplitude phase response when light is applied about 4–6 hours after subjective lights off. We also found that light applied 5 hours after lights off led to greater downregulation of genes than upregulation when sampled 1 hour after light pulse. We additionally identified homologs of 6 canonical clock genes and found unexpected patterns of expression.

## Results

### *M. wittfeldae* has a high amplitude Phase Response Curve

**Fig 1:**
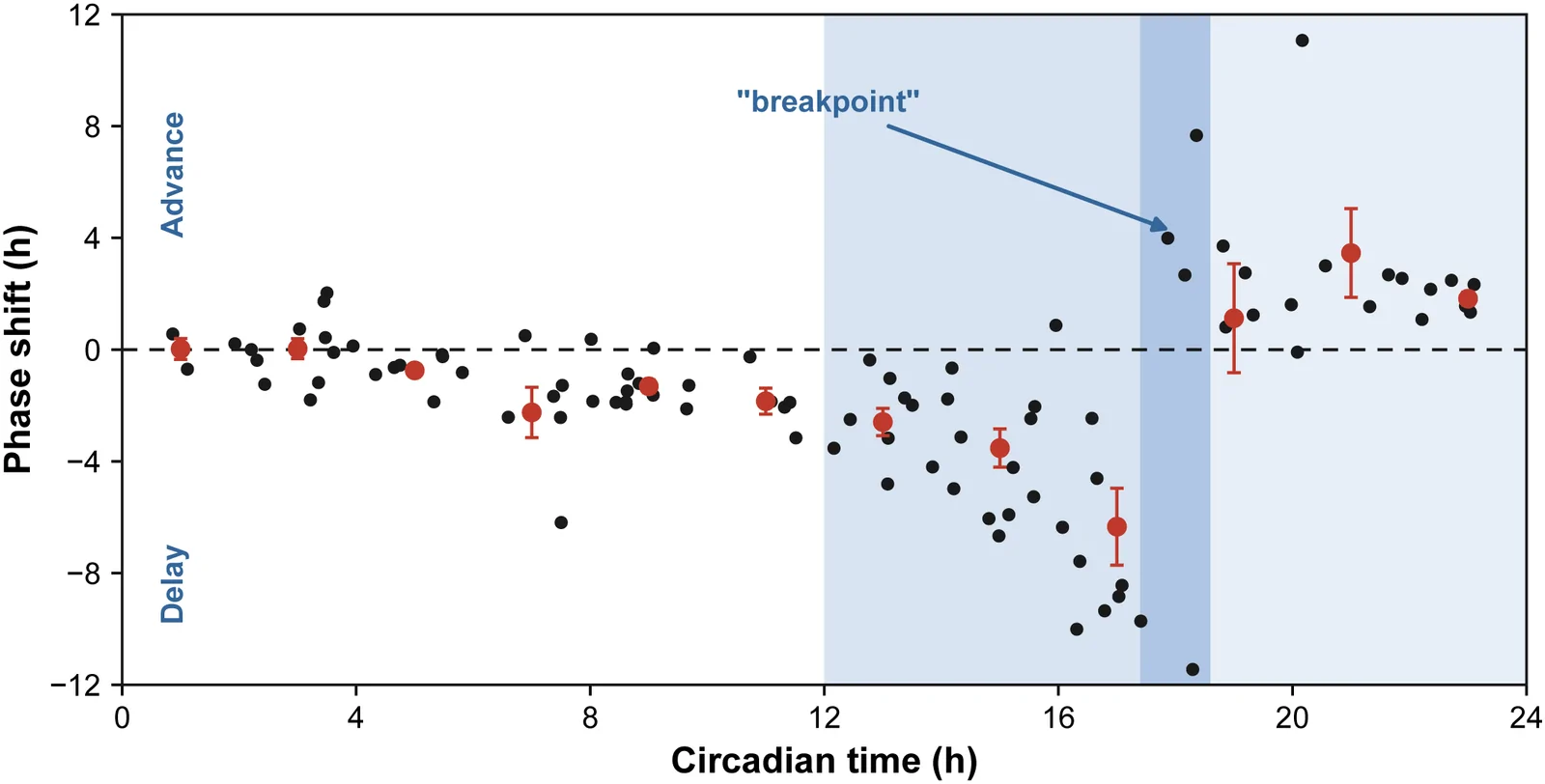
Experimental phase response curve. The phase response curve of locomotor activity of *M. wittfeldae* inferred from the application of one-hour light pulses at different phases in an individual spider’s circadian day. Each point represents the phase change induced by light at that individual’s circadian time. Red dots show mean phase shift ± SEM for 2-hour blocks (n varies by time point: 3, 11, 7, 6, 12, 5, 9, 12, 10, 8, 6, 6, for each respective bin; total n = 95).

To our knowledge, we have derived the first PRC to light for any spider species (Fig 1). Adult female *M. wittfeldae* were wild-caught in northeastern Tennessee between June and August 2017 for 4 separate trials, in which they were kept in an environmental chamber with 12:12 LD for 2–3 days, and then transferred to locomotor-activity monitors at 24 ± 0.5°C for 5–8 days in constant darkness before receiving a single 1-h light pulse from full-spectrum fluorescent tubes. Because spiders were free-running (with variation among individuals in FRP) and the pulse delivery time differed across the four trials, the pulses cumulatively spanned the full circadian cycle. Activity onset was used as the phase reference point (CT 12, following the nocturnal convention) and phase shifts were calculated as the offset between pre- and post-pulse activity-onset regression lines (see Methods for full details).

Of 128 individual spiders, 95 survived the entire duration of the experiment and yielded phase advances or delays in response to a 1-hour light pulse (S2 File: Table S1). There was no significant difference (t-test for paired samples, *P* = 0.14) between the mean FRPs before (23.48 ± 0.07 h) and after (23.40 ± 0.09 h) the light pulse, indicating the absence of light stimulus after-effects on the circadian period. Indicative of a type 0 PRC [53], there are high amplitude (> 6 hr) phase advances and phase delays and an abrupt discontinuity or “breakpoint” at the transition from delays to advances at approximately CT 17–18. In contrast, the predominant PRC found in nature is type 1, in which the phase shifts are relatively small (< 6 hr; often < 2 hr), and the transition from delays to advances along the PRC is continuous [54].

Quantitative criteria for the type-0 classification [53,55] are met by our data (S1 File: Fig S1; S2 File: Tables S1 and S2). The peak-to-peak amplitude of the bin-mean PRC is 9.80 h, with the largest mean phase delay of −6.34 h at CT 17 (95% BCa bootstrap CI −8.20 to −2.57; n = 10) and the largest mean advance of +3.46 h at CT 21 (CI + 1.48 to +7.81; n = 6). The transition from delays to advances is abrupt: bin-mean phase shift changes by 7.46 h between adjacent 2-h CT bins (CT 17 → CT 19), and an ordinary least-squares regression on individual data points within ±3 h of the breakpoint gives a local slope of +2.06 h/h (95% CI + 0.89 to +3.22; R^2^ = 0.35, *P* = 1.27 × 10 ⁻ ^3^; n = 27), markedly steeper than the slope of 1 that would correspond to phase-conserving (S1 File: Fig S1). Nine of the twelve 2-h bin means have 95% bootstrap CIs that exclude zero (S2 File: Table S2).

### PRC suggests time for maximal gene expression response to light

**Fig 2:**
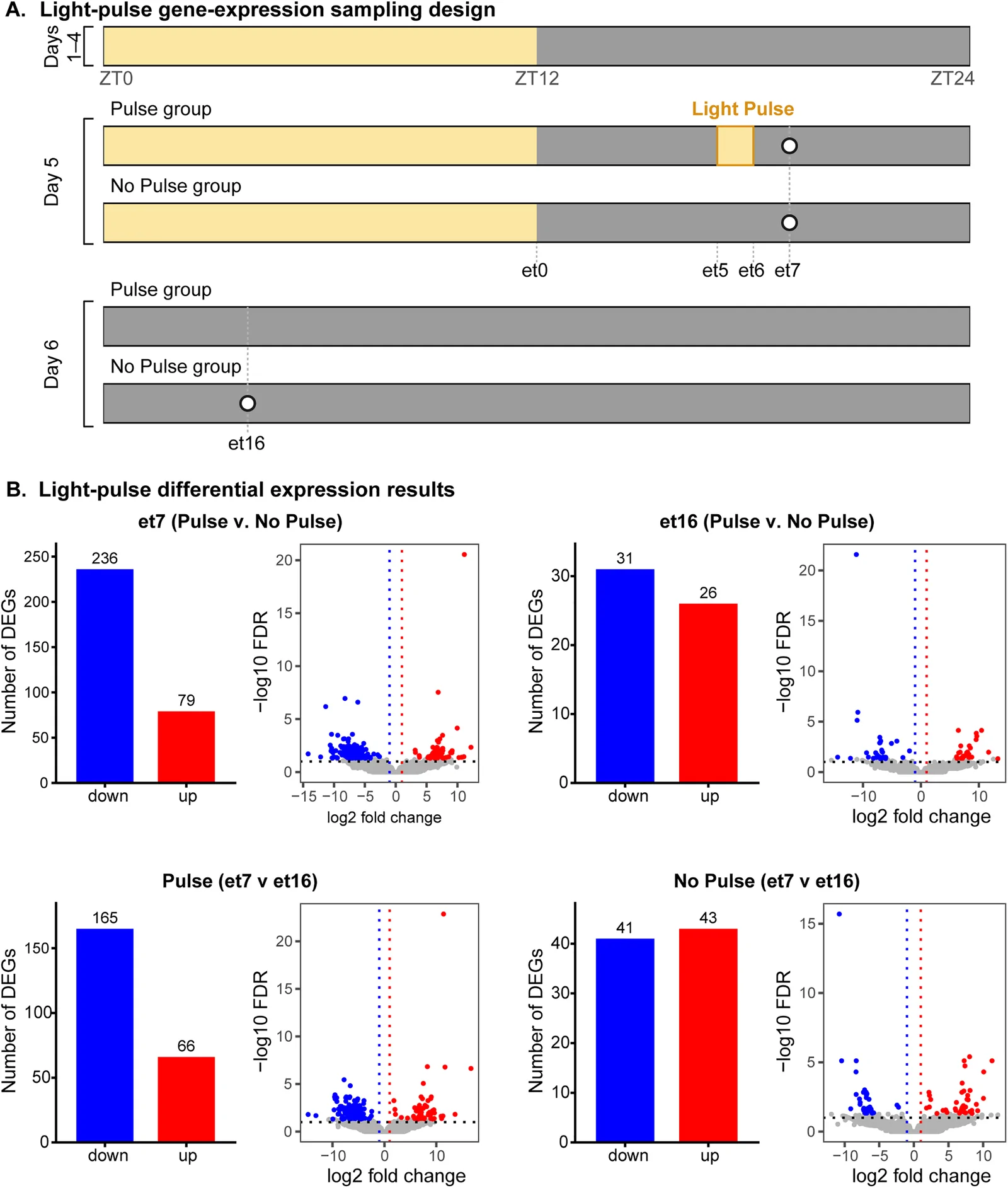
Identification of differentially expressed transcripts associated with light pulse and collection time. (A) Timeline for RNA-sequencing experiment. Spiders were entrained to LD 12:12 for 5 days (ZT0 set to lights on). At the end of the last light period (experimental time 0, et0), half of the subjects were left in constant darkness (No Pulse group), and the other half was exposed to 1 hour long light pulse (Pulse group) 5 hours after darkness onset (et5). The samples were collected in both groups one hour after the end of the light pulse (et7) and 10 hours after the pulse (et16). (B) Volcano plots showing the negative log_10_ of the false discovery rate (-log10 FDR) value (Y-axis) against log_2_ fold change (X-axis) for each of the 4 pairwise comparisons. The down and up-regulated differentially expressed transcripts (DEGs) at a false discovery rate (FDR) < 0.05 with |log_2_ fold change| > 1 are depicted in blue and red, respectively.

The PRC showed that applying a 1-hour light pulse at CT 17–18 caused maximal phase advances and delays in locomotor activity (Fig 1). We reasoned that a 1-hour light pulse at this time should additionally shift the phase of expression for circadian clock or clock-controlled genes. We thus designed an experiment for gene expression analysis in which spiders were entrained to a 12:12 LD cycle for 5 days to synchronize gene expression. On the evening of the 5^th^ day, the lights were turned off (et0, here “et” denotes experimental time, measured in hours after the final lights-off transition at the end of LD entrainment), but one group of 10 spiders received a 1-hour pulse of light (Pulse group) 5 hours after lights off (et5-et6) and the other group of 10 did not (No Pulse group). The timing of the pulse of light is at Zeitgeiber Time (ZT) 17–18, analogous to CT 17–18 (Fig 2A).

Due to resource-limitations, we could only sample for RNA-sequencing at two times after the light pulse. We thus sampled 5 spiders for RNA isolations from Pulse and No Pulse groups 1 hour after the end of the light pulse (et7, or ZT19) and 10 hours after the light pulse (et16, analogous to CT 4) (Fig 2A). With these 2 sampling times, we cannot determine rhythmicity but can identify the acute effects of light on gene expression.

Since no genomic resources exist for *M. wittfeldae*, we *de novo* assembled 266,300 transcripts from the 20 individual RNA-seq libraries, representing 184,077 Trinity-defined “genes”. Annotation of the “genes” resulted in 9 categories of sequences, 4 of which were unlikely to encode proteins and were thus removed prior to further analyses (S1 File: Table S3). Of the retained translated genes, 25,489 were further annotated by significant alignment to a protein in SwissProt and/or PFAM, and 25,127 were assigned Gene Ontology (GO) Terms based on the SwissProt, PFAM, and/or *D. melanogaster* alignments. Comparison with 1066 Benchmarking Universal Single-Copy Orthologs (BUSCO v3.0.2, [56]) from arthropods suggests that our transcriptome is of high quality, with 98.4% (91% as single copy and 7.4% as duplicates) of BUSCO genes represented completely and 0.3% represented by fragmented sequences.

### Light induces short-term down-regulation of genes

Consistent with a large and immediate effect of light on gene expression, the most differentially expressed (DE) genes were observed between Pulse and No Pulse groups at the early collection time (Fig 2B; S1 File: Table S4; S3 File: Tables S5 and S6). Also, consistent with a large effect of light, more genes were differentially expressed between the two collection times in the group that received a light pulse than the group that did not (compare bottom panels in Fig 2B). Additionally, individuals receiving the light pulse and sampled 1 hour later (Pulse, et7) had the most similar expression profiles (S1 File: Fig S2).

We further found that the light pulse was more likely to down-regulate than up-regulate genes (Fig 2B, S1 File: Table S4). The most dramatic shift toward down-regulation was observed at the first collection point, et7 (Fig 2B), where between light pulse and no light pulse 236 transcripts were down-regulated, but only 79 were up-regulated. However, by the time of the second collection, et16, the number of down-regulated genes was very close to that of up-regulated genes (31 vs. 26). Furthermore, for the group experiencing the light pulse, transcript abundance was lower at the early collection time (et7) than at the later collection time (et16) (Fig 2B, 165 down vs 66 up). In contrast, without the light pulse, similar numbers of transcripts were up- and down-regulated between et7 and et16 (Fig 2B, 41 down vs 43 up).

**Fig 3:**
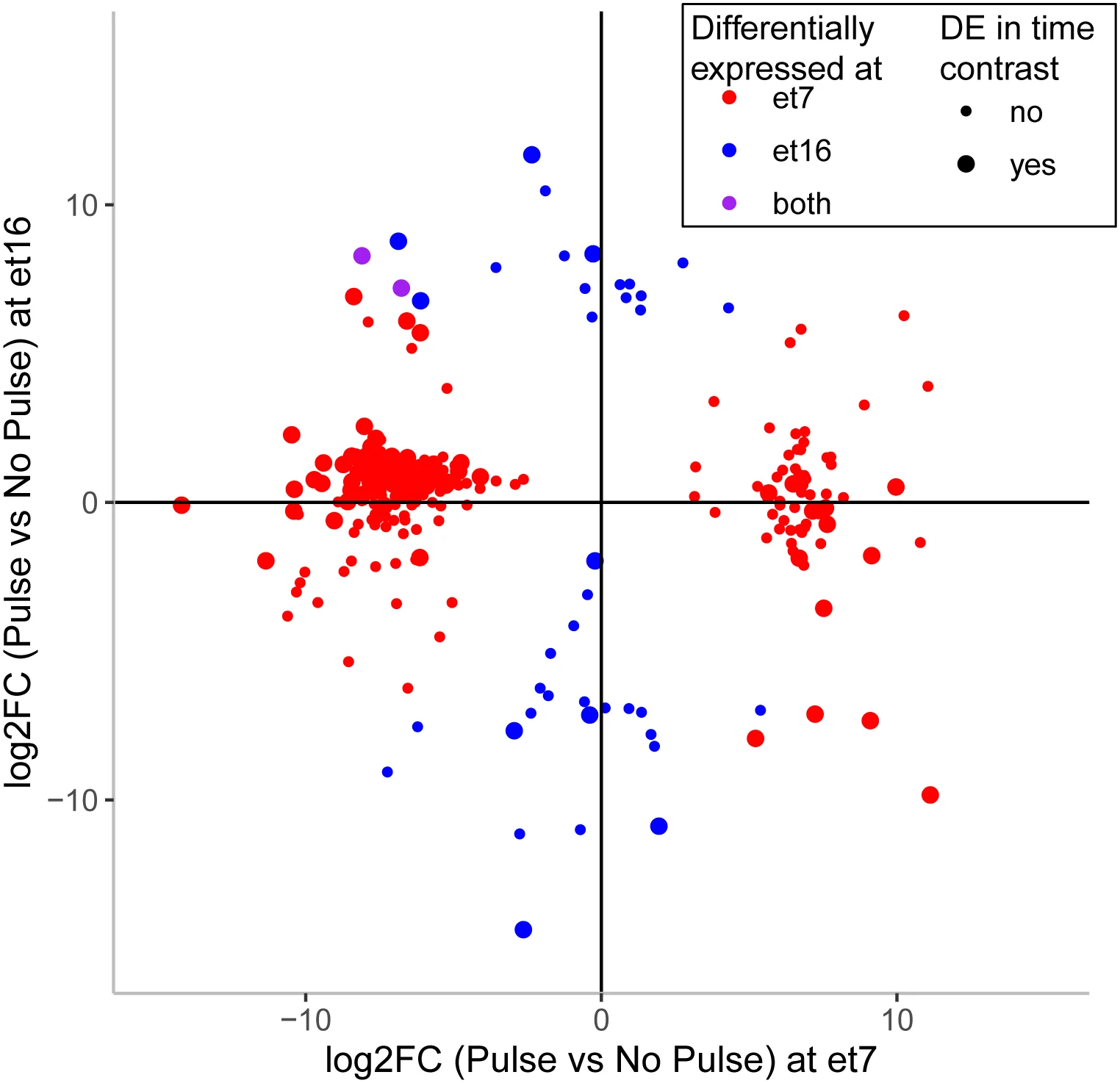
Effect of light on gene expression between collection times. Log_2_ FC of differentially expressed transcripts between Pulse and No Pulse condition at et7 plotted against et16. Transcripts that met the CPM threshold for DE analysis (>0.1 CPM in at least 2 samples) at both times and were DE (FDR < 0.05) at either time are shown. Color indicates the contrast in which each transcript was differentially expressed: transcripts significant only at et7 are shown in red, only at et16 in blue, and at both time points in purple. Circle size indicates whether the transcript was also differentially expressed in at least one of the et7-vs-et16 contrasts (large circles: DE across pulse and time contrasts; small circles: DE in pulse contrasts only; see also S1 File, Fig S3).

To explore how an individual transcript’s response to light changes in the short term versus longer term, we plotted the log_2_ fold change (log_2_ FC) of Pulse/No Pulse at et7 versus et16 for transcripts that were DE at either time (Fig 3). We found that most of the light-downregulated transcripts at et7 were upregulated at et16 (177 of the 220 that also met the Counts Per Million, CPM, threshold for analysis at et16), albeit only 2 significantly so. The light-upregulated genes at et7 were approximately evenly split between up and downregulated at et16, with none significant at the later time. Light responsive DE transcripts at et16 were also evenly split in their earlier expression pattern (Fig 3).

The light pulse also resulted in enrichment of gene functions (S4 File: Tables S7-S10; S1 File: Fig S4). Without a light pulse only three gene ontology (GO) terms were significantly enriched in transcripts differentially expressed between sampling times relative to the whole transcriptome – extracellular space, ion binding, and cell population proliferation. With a light pulse, 8 GO terms were significantly enriched in genes differentially expressed between sampling times, including multiple terms associated with metabolism, cell proliferation, and growth (S4 File: Table S8). Similarly, genes differentially expressed between Pulse and No Pulse at the early sampling time were significantly enriched for 13 GO terms also suggestive of metabolism and cellular growth, as well as cellular transport (S4 File: Table S9). Most of the transcripts associated with enriched GO terms were downregulated in response to light, including cell proliferation Ras-family protein encoding transcripts and the immunoglobulin DSCAM2 encoding transcript (S3 File: Table S5).

### Canonical clock genes are present in spiders but are weakly differentially expressed

**Fig 4:**
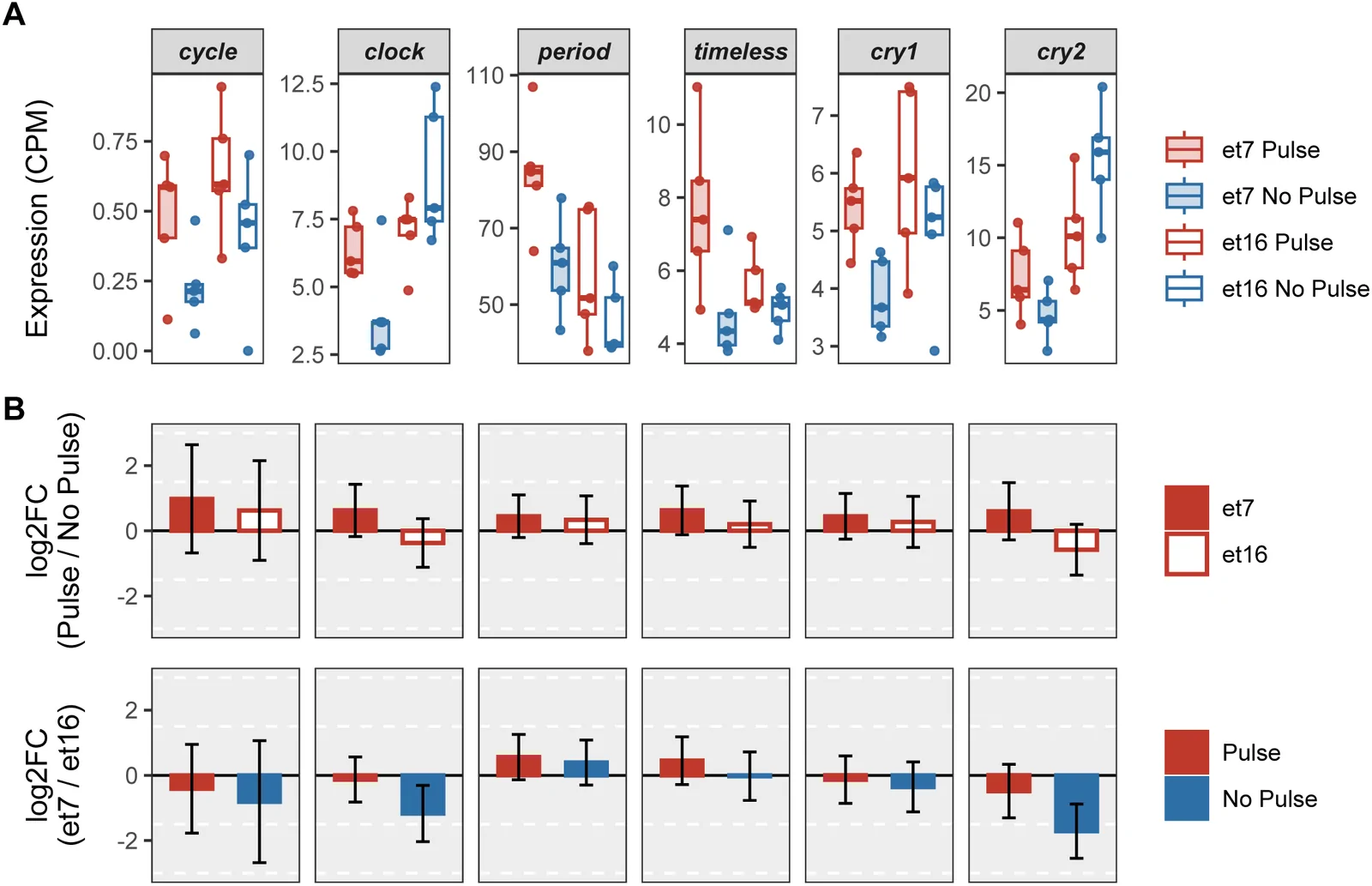
Transcript abundance and differential expression of six canonical clock genes. **(A)** Transcript abundance (edgeR normalized CPM) for each gene. Boxes show the median and interquartile range, whiskers the values within 1.5 × the interquartile range, and points the individual spiders (n = 5 per group); the y-axis is scaled separately for each gene. **(B)** Differential expression (log₂ FC) estimated with edgeR, separated into the effect of the light pulse (top; Pulse vs. No Pulse, with positive values indicating higher expression with the pulse) and the effect of time (bottom; et7 vs. et16, with positive values indicating higher expression at et7). Error bars are approximate 95% confidence intervals of the log₂ fold change derived from the likelihood-ratio statistic. No comparison met the threshold for significance (FDR < 0.05).

We identified orthologs of each of 6 canonical clock genes (*tim, per, cyc, clk, cry1, cry2*) in our *M. wittfeldae* transcriptome using a phylogenetic approach (see Methods for details). Only *cry2* approached significant differential expression in any of our pairwise comparisons. Specifically, *cry2* was more highly expressed at the second collection time (et16) than the first collection time (et7) when not subject to a light pulse (*P* = 0.000055, FDR = 0.054, Fig 4). *Clk* had a similar pattern of expression as *cry2* with lower expression at et7 than et16 (*P* = 0.0078), although none of the comparisons met the FDR < 0.05 threshold for significance (Fig 4).

## Discussion

### First phase response curve for a spider suggests biological mechanisms of their circadian clock

A fundamental property of circadian oscillators is that the internal phase of rhythm is synchronized with the environmental light-dark cycle through the mechanism known as entrainment [57]. To describe the underlying dynamics of entrainment, it is useful to perturb a rhythm with a single stimulus (such as a light pulse) and measure the response in subsequent circadian cycles. This approach, pioneered by Pittendrigh, is described by a nonparametric (discrete) model of entrainment [27]. The model assumes that light at discrete times of day (e.g., dawn and dusk) falls at specific phases of the pacemaker and causes instantaneous phase shifts that are equal to the difference between the period of the rhythm and the period of the LD cycle. The phase response curve (PRC), which is a graphical representation of the shifts in response to stimuli given at different phases, has been extensively used to quantify nonparametric entrainment mechanisms [55,58]. Numerous experiments have described PRCs in multiple model organisms [55] with remarkable similarities in the pattern observed: the response to light typically is largest at time points where the organism is not exposed to light under entrained conditions. In this regard, our experimental PRC demonstrates a similarity to other models described so far: the largest phase shift in response to light occurred during the subjective night, and a “dead zone” was observed during the subjective day.

Two types of PRC can be distinguished: weak resetting PRC (type 1) and strong resetting PRC (type 0). The type 1 PRC has small (typically less than two hours) phase shifts, while type 0 has larger phase shifts [59]. The PRC is considered a property of the circadian pacemaker, and its type depends on the strength of, or sensitivity to, the stimulus [60–62]. It has been demonstrated in several model organisms that an increase in light intensity or light duration can induce a PRC transition from type 1 to type 0 [63]. It is, therefore, surprising that our experiment reveals a strong, type 0, PRC in response to a brief (1 hr.) stimulus of average intensity (~1,400 lux, ~ 14−19 W m^-2^ of broadband visible irradiance). In insects, such light intensity typically results in type 1 (weak) PRC. For example, in cockroach *Nauphoeta cinerea*, ~ 1,600 lux stimulus of 3 hours duration leads to a weak type 1 PRC, and an increase in stimulus duration to 12 hours is needed to switch to type 0 [64]. In the mosquito *Culex pipiens quinquefasciatus*, 2-hour 8,000 lux pulses were needed for transition to type 0 [65]. For *D. melanogaster*, even 12 hours of 3000 lux light pulse still induces type 1 PRC, with the transition to type 0 only possible in *per* mutants [37].

The strength of the light stimulus is not the only potential mechanism to induce a strong, type 0, PRC. The theory of oscillation states that the balance between the stimulus intensity and oscillator amplitude is crucial for PRC shape. That is, the low amplitude (weak) oscillator can produce a large change in response to a moderate stimulus [39]. Therefore, it is possible that if spiders’ clock proteins have low amplitude oscillation, a light pulse of normal amplitude can produce large changes. A low amplitude oscillator could also explain the limited difference in expression levels between time points for the canonical clock genes (Fig 4).

Another possible explanation for the observed type 0 PRC in *M. wittfeldae* might be that light is a very strong stimulus for spiders. In other words, the effect of a single photon of light on a circadian protein in spiders could be more powerful than in insects. In this case, spiders would have evolved a different pathway by which light affects the circadian clock. For example, if the circadian clock is located directly in the eyes, light can affect the clock proteins directly. Examples of entrainment through the eyes have been documented in birds and mollusks [66,67]. In the case of the Japanese quail, the phase response curve is also type 0 [68], suggesting that an ocular circadian clock could increase sensitivity to light. Light could also affect circadian proteins expressed in the brain directly if the carapace is transparent. For instance, *cry1* is expressed in the *D. melanogaster* brain and is directly modified by light [69]. We do not currently have evidence that the *M. wittfeldae* carapace is transparent, but circadian expression in the eyes and/or increased sensitivity of a circadian protein like CRY1 to light could contribute to the observed strength of phase shifting in this species.

Since the activity of *M. wittfeldae* [48] and other spider species [47,49,50] increases dramatically at the onset of darkness, light likely has an inhibitory effect on locomotor activity. Inhibition could be direct (e.g., masking) or act on a step in the circadian molecular clock. Regardless of the neural mechanisms of circadian light response in *M. wittfeldae*, the type 0 PRC is informative about spider entrainment and the potential reasons for the wide variation in FRP among spiders. Since most organisms, including spiders, live in a cycling environment, the phase of entrainment rather than the free-running period should be the main subject of natural selection [70]. The endogenous clock may have evolved to time biological events relative to the external day-night cycle. Thus, the phase angle of entrainment, the fixed time interval between the zeitgeber and the internal clock, could be the trait subject to natural selection [71]. Our previous findings demonstrate that *M. wittfeldae* and two other spider species have sharp activity onset within 30 minutes of lights off while having a wide range of FRP [47–49]. The sharp PRC observed in *M. wittfeldae* and consistent phase angle of entrainment suggest that spiders easily entrain to light, and thus, there may be weak evolutionary pressure to keep FRP close to 24 hours.

### Spiders retain ancestral animal circadian clock genes

We identified homologs of five genes (*per*, *tim*, *clk*, *cyc*, *cry2*) involved in the transcription-translation feedback loop that appears to be conserved across animals [14–16]. We also identified a homolog of the photoreceptor encoding gene (*cry1*) that is primarily responsible for the entrainment of the fruit fly clock. In *M. wittfeldae*, we found *cry2* was almost significantly differentially expressed between our two sampling times when spiders were not subject to a pulse of light (Fig 4), consistent with cycling expression. The pulse of light caused a loss of differential expression between time points, perhaps reflecting a shifted phase of *cry2* expression in response to light. Thus, if confirmed, spiders may retain the ancestral animal clock mechanism in which CRY2 is a transcriptional repressor in the negative feedback loop [72,73].

However, patterns of expression for *cry2* and other clock gene homologs were unexpected. First, none of the other clock gene homologs were differentially expressed between time points. In butterflies and mammals, CRY2 interacts with PER to repress their own transcription, and the expression patterns of *cry2* and *per* are synchronized [72,74–76]. In *M. wittfeldae*, however, *per* expression was consistent across all conditions rather than mirroring the expression pattern of *cry2* (Fig 4). The clock gene homolog with the most similar expression pattern to *cry2* was instead *clk*. In all examined insects and in mammals, CLOCK promotes transcription of *per* and other clock genes when it dimerizes with CYCLE/BMAL1. In fruit flies, *clk* is rhythmically expressed, but with two peaks per day [10], and in honeybees, *clk* expression mirrors the cycling of *per*, *tim*, and *cry2* [72]. On the other hand, many insects and mammals have a constant expression of *clk* throughout the day, and instead, *cyc* is rhythmically expressed [72,77,78]. We found *cyc* was very lowly expressed (< 1 CPM) in all our conditions. Further experiments are needed to determine if this low-level expression produces sufficient CYCLE to dimerize with CLOCK or if CLOCK can promote transcription on its own or with a different partner in spiders.

In fruit flies and potentially other insects, PER interacts with TIM to repress their own transcription and have synchronized expression [75,79,80]. We found that *tim*, similar to *per*, has a pattern of near-constant expression levels across time and light pulse conditions. It is possible that our two time points coincided with rising or falling phases rather than peak and trough (Fig 4). It is also possible that the amplitude of oscillations is low, which would be consistent with the type 0 PRC for *M. wittfeldae*, since low amplitude oscillators are easily perturbed [40,41,81]. However, sampling more times points is needed to establish the rhythmic, or lack of rhythmic, expression of these conserved clock genes.

We were especially interested in discovering the photoreceptor encoding homolog, *cry1*, since spiders easily entrain to light:dark cycles and CRY1 is essential for robust entrainment in fruit flies [17,69,82] and other arthropods [74,83]. *Cry1* transcript abundance did not differ between time points or light pulse conditions. In fruit flies, *cry1* cycles, but light does not directly impact transcription levels of *cry1*. Instead, light changes the conformation of CRY1 to cause degradation of TIM, so it may not be surprising that our pulse of light did not affect the transcript abundance of *cry1*. Like *per* and *tim*, our two sampling times may not have caught the peak and trough of *cry1* expression, *cry1* may not oscillate, or the amplitude of oscillations may be low. Further experiments are needed to determine if *cry1* encodes a photoreceptor in spiders and if it is involved in circadian entrainment.

### Light immediately downregulates many genes and likely affects growth and development

When we applied a light pulse at the time of night when *M. wittfeldae* showed the greatest behavioral sensitivity to light (Fig 1), we found many more genes were downregulated than upregulated 1-hour after the end of the light pulse (Fig 2B). These results are consistent with an inhibitory effect of light on transcription. More than 80% of these significantly downregulated transcripts in short term response to light had a flipped pattern at our later collection time – they were upregulated at et16 in response to light, although only 2 significantly so (Fig 3). This pattern could be consistent with a shifted phase of transcript abundance, as seen for *per* in *D. melanogaster* – when a 1-hour light pulse is applied near the normal peak of the *per* cycle, its mRNA abundance immediately drops below untreated, but a few hours later *per* abundance of the light treated flies is higher than untreated [35]. Since none of the genes in this cluster with flipped expression are homologous to known transcriptional repressors, we do not suggest any of them are regulatory. Instead, they may be outputs of the circadian clock that had a shifted phase of gene expression. With only 2 sampling times, however, other process could explain the flipped pattern of expression. For instance, there could be an overcompensation of transcription to make up for the acute reduction during the light pulse.

The transcripts that responded to light had multiple functions and were distributed throughout the cell (S1 File: Fig S4). Enriched GO-terms associated to transcripts involved in immune response, growth, and development. It has been shown in *D. melanogaster* that the immune response is under circadian control [84,85]. Exposure to night light also leads to reductions in immune function in vertebrates and invertebrates [86,87]. The enriched GO terms are also consistent with prior evidence that exposure to light at night alters circadian rhythms, inhibits reproduction, accelerates development, and leads to increased mortality in vertebrates [86], fireflies [88], and *D. melanogaster* [89]. In an Australian nocturnal araneid spider, exposure to artificial light at night accelerated juvenile development, resulting in spiders progressing through fewer molts [90]. Light intensity also controls the eclosion circadian rhythm in *D. melanogaster* [91]. Our results for *M. wittfeldae* further support a role of light in affecting immune function, development, and reproduction.

## Conclusions

Our results represent the first analysis of a non-insect arthropod Phase Response Curve, which has a strong type 0 shape. The intense phase-shifting response to light could be due to a weak circadian oscillator or to the heightened sensitivity of circadian proteins to light. Patterns of expression of clock gene homologs in spiders may be distinct from other arthropods, and further experiments on these and additional potential cycling genes and proteins could help distinguish the mechanism of spiders’ wide range of FRP and strong entrainment to light.

## Methods

### Experimental phase response curve

Adult female *M. wittfeldae* were wild caught in Washington and Sullivan Counties in northeastern Tennessee, USA in June, July, and August 2017. Permits were not needed since *M. wittfeldae* is not threatened or endangered and collections took place on private docks with the owners’ consent. Orb weaving adult males tend to leave their webs in search of females and the sex ratio at our sampling sites is heavily female biased. We thus restricted sampling to the abundant and easy to find females. Individual spiders were placed in clear 8 oz plastic deli cups in the laboratory, fed crickets, and lightly sprayed with a mist of distilled water. The spiders were kept in an environmental chamber (24 ± 0.5 °C) in their deli cups for 2−3 days with a LD 12:12 hr cycle provided by four vertically mounted full spectrum fluorescent tubes (~1,400 lux corresponding to 14−19 W m⁻^2^ of broadband visible irradiance). The light-dark transition was set at 20:00 hr local time. Individual spiders were then placed in clear glass tubes (25 mm diameter x 15 cm length), and the tubes were inserted into locomotor activity monitors (model LAM 25, Trikinetics Inc., Waltham, MA, USA). The monitors were then placed within the same temperature-controlled (24 ± 0.5 °C) environmental chamber. The activity was recorded continuously for each spider in 1-minute bins via interruptions of infrared beams in the activity monitors. Locomotor-activity monitors were re-used across four trials; the same monitor position on different trial dates therefore corresponds to a different individual spider. Each spider was tested at only one circadian time. Spider identity was uniquely defined as the combination of monitor position and trial date, yielding 95 independent individuals contributing one phase-shift measurement each across the four trials (n = 25 for trial starting 8 Jun 2017; 24 for trial starting 30 Jun 2017; 20 for trial starting 21 Jul 2017; and 26 for trial starting 11 Aug 2017).

Using an Aschoff type 1 protocol [92], a phase response curve was derived from the results of the four trials. Spiders in the monitors were exposed to 5–8 continuous days of constant dark (DD) prior to a 1-hour light pulse (~1,400 lux as in LD). After the light pulse, spiders stayed in monitors for another 6–11 full days of DD. The pulses were delivered at a different time of day in each trial (12:00, 18:00, 6:00, 22:04, respectively). The onset of the pulse was considered the stimulus phase. Because of the broad range of FRPs in this species [48], the pulses delivered in the four trials cumulatively encompassed the entire span of the circadian cycle (Fig 1, S2 File: Table S1). In two trials, the spiders transitioned directly from the LD cycle to DD conditions when placed in monitors. In the other two trials, the spiders were given an additional two or three complete days of LD 12:12 (with the light-to-dark transition at 20:00 hr, as in the other trials) in the monitors before transitioning to DD.

The activity data were displayed as double-plotted actograms for each spider using ClockLab Analysis 6 Software (Actimetrics, Wilmette, IL, USA). To determine the circadian time (CT) at which the light pulse occurred for individual spiders, activity onsets were used as the phase reference points, with activity onset denoted as circadian time, CT 12. This is consistent with nocturnal organisms that become active at dusk. *M. wittfeldae* is highly nocturnal with locomotor activity beginning shortly after the light-to-dark transition [48]. Phase shifts were calculated by tracking the pre- and post-pulse FRPs using the linear regression function in ClockLab through activity onsets for each individual spider. A t-test for paired samples was used to determine if there was a significant change in FRP following the light pulse.

Quantitative criteria for the type-0 PRC classification followed Winfree (2001) and Johnson (1999) [53,55]. For each 2-h CT bin, we computed the mean phase shift, standard deviation, range, parametric (Student’s t) 95% confidence interval, and bias-corrected and accelerated (BCa) bootstrap 95% confidence interval (10,000 bootstrap iterations) (S2 File: Table S2). The slope of the PRC through the delay-to-advance breakpoint was estimated by ordinary least-squares regression on individual data points within ±3 h of the bin-mean discontinuity, with the 95% CI on the slope computed from the regression standard error. Analyses were implemented in Python 3 (NumPy, SciPy, pandas, matplotlib) on the per-spider PRC dataset with each row indexed by a Spider ID derived from monitor position and trial date (S2 File: Table S1).

### Gene expression experimental design

Adult female *M. wittfeldae* were collected on 16 July 2018 in Washington County, TN, and placed in clear plastic deli jars on a lab bench receiving natural lighting. All spiders were provided with a wet cotton ball and offered a small cricket. On 18 July 2018, each spider was moved to a clear glass or plastic tube (25 mm diameter x 15 cm length) and inserted into a locomotor activity monitor (model LAM25, Trikinetics Inc., Waltham, Massachusetts, U.S.A.). The monitors were placed into two separate temperature-controlled (24 ± 0.5 °C) environmental chambers with the lights on (1,400–1,600 lux, ~ 14–17 W m ⁻ ² of broadband visible irradiance) at ~16:00 until 20:00, after which the spiders remained in the dark for 3.5 days (84 hours) to establish the free-running period of each individual. In order to synchronize the circadian rhythms of the individual spiders, they were then entrained to a 12:12 LD cycle for five days. On the evening of the fifth day, the lights went out at 20:00 (et0), as per the prior 4 days. However, one chamber received 1 hour of light (1,400–1,600 lux) starting 5 hours after the lights were turned off (et5-et6, 01:00–02:00 27 July 2018) (Pulse group). The other chamber remained dark (No Pulse group). One hour after the end of the light pulse (et7, 03:00) 5 spiders were removed from each environmental chamber and snap frozen in liquid nitrogen under dim red light. Both chambers remained dark until the final collection time 9 hours later (et16, 12:00), when five spiders were again removed from each chamber and snap frozen in liquid nitrogen under dim red light.

### RNA-sequencing, transcriptome assembly, and annotation

Total RNA was isolated from one half of a cephalothorax (the fused head-body) of each of the 20 experimental spiders by homogenizing tissue in TRIzol (Invitrogen) and further purifying with the RNeasy Mini Kit (Qiagen) with on-column DNaseI digestion to remove contaminating DNA. RNA quality and quantity were verified with the Agilent 2100 Bioanalyzer, and 20 individually barcoded cDNA libraries were constructed with the TruSeq kit (Illumina) by the Genomics Research Laboratory at the Biocomplexity Institute, Virginia Tech. All 20 libraries were multiplexed and sequenced in a single lane with the NextSeq 500 (Illumina) using the High Output mode with 300 cycles (150 base paired end reads). De-multiplexing and barcode removal were performed at Virginia Tech.

Transcripts were *de novo* assembled with Trinity v2.8.4 [93] from all 20 RNAseq libraries. Raw reads were trimmed of low-quality base calls using Trimmomatic [94], and *in silico* normalization was used to reduce the number of reads entering the assembly phase, using scripts in Trinity.

Annotation involved a multi-step process using the Transcriptome Trimming and Annotation Pipeline (TrTAP) [95]. Trimming started when any transcripts with a significant BLASTN match to the SILVA ribosomal database v.132 [96] or a tRNAScan v.1.3.1 match [97] were identified (RNA) and removed from further analysis. Second, all the transcripts were compared to a custom-made database of spider silk proteins encoded by the spidroin gene family [98] and subsequently to the proteomes of 5 arthropod species using BLASTx. These species included 3 spiders (listed from most closely related to least): *Araneus ventricosus* (GCA_013235015.1_Ave_3.0, [99])), *Trichonephila clavipes* (MWRG00000000.1, [100]), and *Stegodyphus mimosarum* (GCA_000611955, [101]); another arachnid, *Ixodes scapularis* (GCF_016920785.1, [102]); and an insect, *D. melanogaster* (FB2019_08 [103]). The *D. melanogaster* gene matches with a cutoff of 1e-40 was used to identify chimeric sequences with a custom Python script described in [104].

For each Trinity “gene”, the transcript with the best BLAST alignment, as determined by the highest bit-score, or with the longest open reading frame (ORF) if there were no BLAST alignments, was chosen to represent that gene, with all the genes used to calculate the expression with RSEM [105]. To further reduce redundancy and identify fragments of genes, the best matching gene of each protein in the curated silk gene database plus the 5 species proteomes were also identified. These reciprocal best matches were always retained for downstream analyses (BEST, S1 File: Table S3). If a Trinity gene was not the best match of a database protein, it was considered “GOOD” if it aligned to >20% of the database protein and exceeded 1 TPM (transcript per million) in at least one RNAseq library. Genes that aligned to >20% of the database protein and did not meet the 1 TPM threshold were dubbed “LOW_EXP” (S1 File: Table S3). Genes that aligned to <20% of the database proteins were dubbed “LOW_COV” (S1 File: Table S3). For the genes with no BLAST alignments to a proteome, the gene was called “LONGORF” if the ORF exceeded 50 amino acids and had > 1 TPM in at least one RNAseq library (S1 File: Table S3). ORFs that exceeded 50 amino acids but had < 1 TPM were called “LOW_EXP_LONGORF” (S1 File: Table S3). Genes that did not have a BLAST hit and an ORF of less than 50 aa were called “NO_HIT”. A reduced set of probable protein-coding genes (BEST, GOOD, LONGORF, and LOWEXP) was then translated using the direction and frame of the matching BLASTX hit or, in cases without such hit, translated according to the longest open reading frame. These translated genes were further annotated by comparison to SwissProt with BLASTP and to PFAM with HMMER v 3.2.1 [106] with Gene Ontology (GO) terms [107,108] assigned to each “gene” based on the best alignment to *D. melanogaster*, SwissProt, and PFAM, with GO SLIM annotations obtained from GO SLIM viewer [109]. The completeness of the reduced transcriptome was assessed with the set of single-copy orthologous genes in arthropods (BUSCO v3, [56]).

### Canonical circadian clock gene identification

We used a phylogenetic approach to determine which transcript was an ortholog of the canonical clock gene in *D. melanogaster* or *Apis mellifera*: *clk*, *cyc/bmal1*, *tim*, *per*, *cry1*, *cry2*. We first used Orthofinder v2.4.1 [110] to identify groups of homologous genes among 6 spider species, a tick, and two insects. In addition to our *M. wittfeldae* transcriptome, we used the predicted proteomes from published transcriptomes or genomes for the following species: *Araneus ventricosus* (Araneidae, BGPR01000000.1), *Trichonephila clavipes* (Araneidae, MWRG01000000.1), *Latrodectus hesperus* (Theridiidae, GBJN01000000.1), *P. tepidariorum* (Theridiidae, GCF_000365465.3), *Stegodyphus mimosarum* (Eresidae, GCA_000611955.2), *I. scapularis* (Ixodidae, GCF_016920785.1), *D. melanogaster* (Drosophilidae, FB2019_08), *A. mellifera* (Apidae, GCF_003254395.1). We then identified the “orthogroups” containing the canonical circadian gene of *D. melanogaster* or *A. mellifera* (S1 File: Table S11). For 5 of the genes, only a single *M. wittfeldae* transcript was placed in the orthogroup; these were considered the ortholog of the circadian gene. For the cryptochromes (*cry1* and *cry2*), we inferred a phylogenetic tree using maximum likelihood (RAxML, [111]). From the resulting tree, we chose the single *M. wittfeldae* transcript in the clade with the canonical circadian clock gene (S1 File: Fig S5).

### Differential gene expression analysis

Differential gene expression among experimental groups was based on gene-level expression estimates derived from transcript abundance estimates as recommended by Soneson et al. [112]. In brief, the expected read counts of each Trinity-assembled transcript were calculated by RSEM v.1.3.1 [105], which takes into account read-mapping ambiguity due to multiple isoforms or even alleles having been assembled. The gene-level counts were then calculated as the sum of the included transcripts, weighted by their length as described in Soneson et al. [112] and implemented through Trinity v2.8.4. Because RSEM can distribute one read among multiple transcripts, some counts were not integer values. These values were rounded for input into differential expression analyses, which require integer values for read counts. Prior to further expression analyses, genes unlikely to encode proteins were removed (S1 File: Table S3). For comparing expression levels among genes and between groups, we used Counts Per Million total counts (CPM) normalized for differences in sequencing depth among libraries with the Trimmed Mean of M (TMM) values calculated with EdgeR, initially with v3.3.6 and later with v4.0 [113].

Four different pairwise gene expression analyses were performed with EdgeR [113]. Our initial analyses were performed in R v4.1.1, edgeR v3.3.6, and then redone with R v4.5.2, edgeR v4.0 (Bioconductor v3.2.2). EdgeR models read counts with a negative binomial distribution, estimates gene-wise dispersions by Cox–Reid adjusted profile likelihood (sharing information across genes), and tests for differential expression between groups by fitting negative binomial generalized linear models followed by likelihood ratio tests. edgeR 4.0 replaced its dispersion and GLM fitting algorithms, but we used “legacy=TRUE” to retain the v3 method. Our four pairwise comparisons were: 1) comparing samples with light pulse to samples without light pulse at et7; 2) comparing samples with light pulse to samples without light pulse at et16; 3) comparing samples collected at et7 to samples collected at et16 with a light pulse; 4) comparing samples collected at et7 to samples collected at et16 without a light pulse. A gene was kept in the analysis if it had sufficient expression (CPM > 0.1) in at least two samples. The significantly differentially expressed genes in the pairwise comparisons were identified with both a false discovery rate (FDR) < 0.05 and an absolute value of log_2_ FC > 1 (S3 File: Tables S5-S6).

### Gene Ontology analysis

GO analysis was conducted to determine the functions enriched in DE genes relative to all annotated genes using the ‘GOseq’ Bioconductor package [114] based on Wallenius non-central hyper-geometric distribution. The lists of GO terms, along with their p-values generated from ‘Goseq’, were summarized and visualized by the REViGO online tool [115] (S1 File: Fig S4).

## Supporting information

S1 File The file includes Figs S1-S5 and Tables S3, S4, S11. (DOCX)

S2 File Includes Tables S1-S2, which provide the per-individual phase response data (S1) and the CT-bin summary statistics (S2). (XLSX)

S3 File Includes Tables S5-S6, which describe the differentially expressed transcripts identified in each of the four pairwise comparisons. (XLSX)

S4 File Includes Tables S7-S10, which describe the GO terms enriched for differentially expressed transcripts. (XLSX)
