# Supplementary material for: Phase response curve and RNA-sequencing demonstrate spiders’ sensitivity to light and pinpoint candidate light-responsive genes
Source: PLoS One. 2026 Sep 23;21(9):e0344063. doi: 10.1371/journal.pone.0344063 (PMC13600489; doi:10.1371/journal.pone.0344063)
Supplement: Supplementary file 1 [file pone.0344063.s001.docx]

**Fig S1.** Phase response curve, type 0 diagnostic. See S1 File: Tables S1-S2 for details of data.

**Fig S2:** Multidimensional scaling (MDS) plots of transcriptomic responses represented in log_2_ Fold Change (log2 FC) with different light pulse treatments and collection time. Distances between samples correspond to leading biological coefficient of variation (BCV) between those samples.

**Fig S3. Cluster of transcripts in response to collection time and light pulse.** Scatterplot circles indicate log_2_ FC values of differentially expressed genes (FDR < 0.05) in either the et7/et 16 Pulse or et7/et16 No Pulse comparisons. Transcripts differentially expressed only with a light pulse are red circles, while transcripts differentially expressed only without a light pulse are blue circles. Two genes are differentially expressed with and without a light pulse, colored purple. The larger circles indicate genes identified as differentially expressed in one of the Pulse/No Pulse analyses (see Figure 3 in main document).

**Fig S4:** REVIGO scatterplots of the enriched GO cluster representatives related to biological processes. The resulting lists of GO terms (Supplementary File 4: Tables S10-S13) along with their p-values (*P* < 0.05) were summarized and visualized by REVIGO reduction analysis tool. (**A**) Significantly enriched GO terms related to biological processes with light pulse vs without light pulse at et7. (**B**) Significantly enriched GO terms related to biological processes at et7 vs. et16 with a light pulse (Pulse). et16 (pulse vs. no pulse) and the et7-vs-et16 no-pulse comparison are not shown because fewer than two biological-process terms were significantly enriched. GO terms are represented by bubbles and are clustered according to their semantic similarities to other GO terms in the gene ontology (adjoining circles are most closely related). Bubble color indicates log_10_ *P*-value for the enrichment, and the two ends of the colors are red and blue, depicting lower- and higher p-values respectively. Bubble size indicates the frequency of the GO term in the underlying reference EBI GOA database (Huntley et al. 2015) (bubbles of more general terms are larger). The semantically similar terms of the identified GO terms were removed using SimRel semantic clustering. Scatterplots of the remaining terms after the redundancy reduction were generated using the source code from REViGO, which plotted the terms in a two-dimensional space by applying multidimensional scaling to a matrix of the GO terms' semantic similarities (Supek et al. 2011).

**Fig S5:** Maximum likelihood tree for Orthogroup containing cryptochromes. The *D. melanogaster* CRY1 protein and its *M. wittfeldae* ortholog are colored red. The *A. mellifera* CRY2 protein and its *M. wittfeldae* ortholog are colored blue. Protein sequences are derived from *M. witteldae* (MW), *Araneus ventricosus* (GBM), *Trichonephila clavipes* (NC), *Parasteatoda tepidariorum* (PT), *Latrodectus hesperus* (LH), *Stegodyphus mimosarum* (SM), *Ixodes scapularis* (IS), *Apis mellifera* (XP), and *D. melanogaster* (FB).

| **Table S3. Annotation of the *de novo* assembly of *Metazygia wittfeldae* with counts for each category.** | |
| --- | --- |
| **BEST**: has a reciprocal best BLASTx alignment to a protein in a reference database | 26114 |
| **GOOD**: has BLASTx alignment to a protein in one of the reference databases but another Trinity gene has a better BLAST alignment | 9655 |
| **LONGORF**: predicted to encode a protein at least 50 amino acid long, but does not have a significant BLASTx alignment to any of the reference proteins | 46325 |
| **LOW_EXP**: same as GOOD, but with < 1 RSEM estimated TPM in any library | 10953 |
| **LOW_EXP_LONGORF**: same as LONGORF, but with < 1 RSEM estimated TPM in any library | 42737 |
| **CHIMERA**: potential assembly of two different protein coding genes because of a very good alignment to different proteins in the reference databases | 48 |
| **LOW_COV**: same as GOOD, except that it only aligns to < 20% of the database protein | 7881 |
| **RNA**: aligns to ribosomal or transfer RNA | 210 |
| **NO_HIT**: has no BLAST alignment and is not predicted to encode a protein > 50 amino acids | 40153 |
| Counts are based on the Trinity-defined “gene”. The bottom 4 categories were retained for read mapping and estimation of expression levels with RSEM, but were removed prior to differential expression analyses. TPM = RSEM (Li and Dewey 2011) estimated Transcripts Per Million mapped transcripts. | |

| **Table S4. Differentially expressed genes.** | | |
| --- | --- | --- |
| Comparison | # DE genes (FDR < 0.05) | # genes examined |
| Pulse vs. No Pulse at et7 | 315 (236 down, 79 up) | 90341 |
| Pulse vs. No Pulse at et16 | 57 (31 down, 26 up) | 102817 |
| et7 vs. et16 with Pulse | 231 (165 down, 66 up) | 100103 |
| et7 vs. et16 with No Pulse | 84 (41 down, 43 up) | 94062 |
| Four comparisons were performed (Supplementary Tables S3-S6) to identify differentially expressed genes (false discovery rate, FDR < 0.05, \|log fold change\|>1) with edgeR (Robinson et al. 2010; Robinson and Oshlack 2010), focusing only on genes with > 0.1 CPM in at least two libraries. Spiders received a 1-hour pulse of light 5 hours after lights off (Pulse) or did not (No Pulse) and were collected 1 hour (et7) or 10 hours (et16) after the end of the light pulse. | | |

| **Table S11. Circadian “clock” gene identification.** | | | |
| --- | --- | --- | --- |
| ID | *Drosophila* Gene(s) |  | *Metazygia* Gene |
| Cycle | **FBpp0074693** |  | MW_TRINITY_DN59140_c0_g1 |
| Clock | **FBpp0076500** FBpp0099478 FBpp0099480 FBpp0306709 FBpp0306710 |  | MW_TRINITY_DN2943_c0_g1 |
| Period | **FBpp0070455** FBpp0304590 |  | MW_TRINITY_DN146_c0_g1 |
| Timeless | **DM_FBpp0077256,** DM_FBpp0305450, DM_FBpp0305451, DM_FBpp0305452, DM_FBpp0305453, DM_FBpp0305454, DM_FBpp0305456, DM_FBpp0305457, DM_FBpp0401565, DM_FBpp0428305 |  | MW_TRINITY_DN7506_c0_g1 |
| CRY1 | FBpp0080934 FBpp0080935 **FBpp0083150** FBpp0307793 FBpp0307794 |  | MW_TRINITY_DN18046_c0_g1 **MW_TRINITY_DN2770_c0_g1** MW_TRINITY_DN308_c1_g1 |
| CRY2 | *Apis mellifera* protein: <https://www.uniprot.org/uniprot/A4GKG5> |  | MW_TRINITY_DN18046_c0_g1 MW_TRINITY_DN2770_c0_g1 **MW_TRINITY_DN308_c1_g1** |
| The circadian genes (bold) were identified for *D. melanogaster* from FlyBase v2019_08. In the case where more than one *M. wittfeldae* transcript was in an orthogroup with a circadian gene, phylogenetic trees were examined for orthology (Figure S5). The ortholog is bolded. | | | |

**References**

Huntley RP, Sawford T, Mutowo-Meullenet P, Shypitsyna A, Bonilla C, Martin MJ, O’Donovan C. 2015. The GOA database: Gene Ontology annotation updates for 2015. Nucleic Acids Res. 43(D1):D1057–D1063. https://doi.org/10.1093/nar/gku1113

Leloup J-C, Gonze D, Goldbeter A. 1999. Limit cycle models for circadian rhythms based on transcriptional regulation in *Drosophila* and *Neurospora*. J Biol Rhythms. 14(6):433–448. https://doi.org/10.1177/074873099129000948

Li B, Dewey CN. 2011. RSEM: accurate transcript quantification from RNA-Seq data with or without a reference genome. BMC Bioinformatics. 12(1):323. https://doi.org/10.1186/1471-2105-12-323

Robinson MD, McCarthy DJ, Smyth GK. 2010. edgeR: a Bioconductor package for differential expression analysis of digital gene expression data. Bioinformatics. 26(1):139–140. https://doi.org/10.1093/bioinformatics/btp616

Robinson MD, Oshlack A. 2010. A scaling normalization method for differential expression analysis of RNA-seq data. Genome Biol. 11(3):R25. https://doi.org/10.1186/gb-2010-11-3-r25

Supek F, Bošnjak M, Škunca N, Šmuc T. 2011. REVIGO summarizes and visualizes long lists of gene ontology terms. PLOS ONE. 6(7):e21800. https://doi.org/10.1371/journal.pone.0021800

Ueda HR, Hagiwara M, Kitano H. 2001. Robust oscillations within the interlocked feedback model of *Drosophila* circadian rhythm. J Theor Biol. 210(4):401–406. https://doi.org/10.1006/jtbi.2000.2226

Wager-Smith K, Kay SA. 2000. Circadian rhythm genetics: from flies to mice to humans. Nat Genet. 26(1):23–27. https://doi.org/10.1038/79134
