# Supplementary material for: Phase response curve and RNA-sequencing demonstrate spiders’ sensitivity to light and pinpoint candidate light-responsive genes
Source: PLoS One. 2026 Sep 23;21(9):e0344063. doi: 10.1371/journal.pone.0344063 (PMC13600489; doi:10.1371/journal.pone.0344063)
Supplement: Supplementary file 2 [file pone.0344063.s006.docx]

**Response to Editor and Reviewers Comments**

Below we provide a copy of the comments (black text, Arial Font) followed by our response (indented blue text). Unless otherwise indicated, line numbers stated in our responses reference the revised manuscript without tracked changes.

**Editor Foulkes**:
Thank you for submitting your manuscript to PLOS ONE. After careful consideration, we feel that it has merit but does not fully meet PLOS ONE’s publication criteria as it currently stands. Therefore, we invite you to submit a revised version of the manuscript that addresses the points raised during the review process.

While all three reviewers appreciated the value and novelty of this PRC analysis for a species of spider, two reviewers (and myself), raise significant concerns regarding the interpretation of the transcriptome results and in particular the merits of the statements made regarding potential links between gene expression changes and the PRC results. For this reason, I would strongly advise the authors to rewrite the transcriptome section of the paper, paying particular attention to the comments and advice of Reviewer #3. In addition, comments raised by all reviewers regarding the reporting and interpretation of the PRC should be addressed appropriately.

Response: Thank you for the opportunity to revise and resubmit. We have added raw data and extended quantification for our phase response curve (PRC). We opted to completely remove the mathematical models and substantially modified text in the Results section to avoid circadian interpretations of gene expression patterns. We retained some circadian interpretations of transcript abundance in the discussion but provide an extended explanation of alternative interpretations and the limitations of sampling only 2 time points. We are glad to be able to retain reference to light responsive genes. We believe our substantial revisions have considerably improved the manuscript. Details of our revisions are described below in response to each of the three reviewers’ comments.

We should point out that in updating figures to address reviewer comments, we re-did the DE analyses in new versions of R, edgeR, and Bioconductor. While we tried to maintain the legacy settings to the extent possible, some changes to edgeR resulted in slightly modified total counts of differentially expressed genes. The updated DE counts did not affect the main patterns of greater downregulation in immediate response to light, with ameliorated differential expression 10 hours later. We have added a description of the versions used to the Methods.

We should also point out than in preparing to submit raw behavior files that are output by the Trikinetics monitors, we could not find a raw file associated with 7 spiders that were originally included in our calculations of the PRC. We thus reanalyzed the 95 spiders for which we had raw files and estimates for phase response (S2 File). The resulting PRC is nearly identical to the original one reported for 102 spiders.

**Reviewer #1**: This manuscript investigates circadian light entrainment in the orb-weaving spider Metazygia wittfeldae using two complementary approaches: the construction of a phase response curve (PRC) based on 1-hour light pulses provided at different circadian times, and RNA sequencing following a light pulse at CT17 to identify candidate light-responsive genes. The authors report a high-amplitude type 0 PRC characterized by strong phase advances and delays and identify differentially expressed genes at both 1 and 10 hours after the pulse. The integration of behavioural assays and transcriptomic analysis in a non-model arthropod represents a key strength of the study and provides novel data to the field.
The manuscript is generally well written, but I would currently recommend addressing the comments reported below.

1. The authors describe a “type 0 PRC” characterized by high-amplitude phase shifts (>6 h) and a breakpoint at CT17. While this is a potentially important result, further clarification and justification are needed to support this classification. Type 0 PRCs are typically associated with strong resetting stimuli. The manuscript would benefit from more explicit quantitative criteria used to classify the PRC as type 0, as well as a clearer description of whether the transitions between delay and advance phase shifts are sufficiently abrupt to meet canonical definitions.

Response: Thank you for the positive reception to the integrated approach to a non-model organism! Thank you also for the opportunity to justify the type 0 classification. We have added text to the Results (lines 154-163) and Methods (lines 455-463) describing how the phase response was classified and showing quantification of the high amplitude type 0 PRC. We have also added a supplementary figure (S1 File: Fig S1) and file (S2 File: Table S2) further demonstrating the high amplitude response and break point between phase delays and advances.

2. The distinction between type 0 and type 1 PRCs can depend on light intensity. How did you choose the light intensity? Did you test other intensities? The authors used 1,400 lux, stating that it is comparable to sunlight in the early afternoon of an overcast day. However, 1,400 lux is much dimmer than typical daylight, even under overcast conditions. It more likely corresponds to illumination under heavy cloud cover or during early morning or late afternoon conditions.
I understand that many published studies on this topic use lux as a measure of illumination, but this is not ideal because it is based on human visual sensitivity. While it may be acceptable for historical reasons and to allow comparisons across studies, I suggest also reporting irradiance values in physical units (e.g., W/m² or µW/cm²).

Response: Thank you for the opportunity to clarify light levels. We have added irradiance values to any place in the text that referenced lux. We have also deleted reference to the light levels being comparable to an overcast afternoon.

3. The authors should clarify key aspects of the experimental design and replication for the behavioural assays. Although more than 100 spiders were used in total, it is important to specify how many individuals were tested for each CT and whether the same individuals were reused across conditions. It should also be clarified whether all measurements are independent.
How consistent were the phase shifts across individuals, given the expected inter-individual variability?

Response: Thank you for the opportunity to clarify the PRC experimental design. Each individual spider was only used for one phase response data point. No spiders were reused. We have added a supplementary file describing each individual’s FRP before and after the light pulse, the time of the light pulse, the CT of the individual, and the phase response of the individual (S2 File: Table S1). We also provide a table summarizing the 2-hour CT bins including number of individuals per bin, and mean, median, range, and confidence intervals of phase shifts for each 2-h bin (S2 File: Table S2).

4. What does the abbreviation “et” mean? While ZT is the standard for entrained rhythms, I am not familiar with “et.” Does it refer to “entrainment tme” or “endogenous time”? In addition, the abbreviation “FRP” for free-running period is not commonly used.

Response: Thank you for pointing out that we had accidentally deleted our definition of “et”, which stands for “experimental time” – with experimental time 0 set to lights off. For our gene expression experiment, we struggled with choice of ZT versus CT, since the first 12 hours after lights off is rightly ‘ZT’, but the next 12 hours could be considered ‘CT’. To avoid confusion, we decided to set the experimental time frame equal to 0 when lights were turned off. We have now defined ‘et’ on first use (line 170 in Results). We also defined the abbreviation FRP on first use (line 65).

5. The observation that genes downregulated one hour after the light pulse become upregulated nine hours later is intriguing. While this pattern is consistent with a phase shift, it does not constitute definitive proof, as it may also reflect transient stress responses or general light-induced pathways.
The authors should avoid language implying mechanistic confirmation and instead frame these findings as the identification of candidate light-responsive genes rather than genes directly underlying entrainment mechanisms.
More broadly, it remains unclear whether the observed gene expression changes reflect a true circadian phase shift or simply acute photic responses. Ideally, sampling across additional time points over a full circadian cycle would help resolve this distinction. If this is not feasible, the limitation should be explicitly acknowledged in the text.

Response: Thank you for the interest in the switched pattern of gene expression. We wish we had the resources to sample additional time points. We agree that multiple processes could have caused the observed pattern, which we had originally stated, but probably not strongly enough. We have now dramatically reduced the text regarding the flipped pattern of expression, limited our discussion of the possibility of a phase shift to a short part of the discussion with reference to experimental mRNA phase shifting by light in flies rather than referencing the models (lines 384-387) and described an alternative explanation for the pattern (lines 390-392).

6. In the paragraph describing the differential expression of clock genes, the authors state that some genes are much more highly expressed than others, but no statistical analysis is reported. This information is necessary to support such claims. In addition, it is unclear whether the data shown in the figure represent means. If so, standard deviations (or other measures of variability) are missing. If they are not means, does each value correspond to a single measurement? The figure legend is not clear and should be more explicit.

Response: Figure 4 in the original manuscript described the log_2_ fold change in each of our 4 pairwise statistical tests for differential expression. We had referenced supplementary tables for p-values, but now have added the confidence intervals to the figure along with additional explanation of FDRs in the text of Results and in the figure legend. The overall expression levels of the clock genes were originally reported in a supplementary figure. However, we agree that it would be useful to include the evidence for expression levels in the main document and have thus updated Figure 4 to additionally include CPM values for the 6 clock genes in each of our 4 conditions.

We should additionally note that in revising this figure, we re-ran the DE analysis using the most recent version of edgeR, which resulted in a few changes to p-values and FDRs, changing slightly the number of transcripts that met the FDR < 0.05 threshold. The clock gene, *cry2*, was one of the transcripts with a slight change in FDR from just under 0.05 to just above 0.05 in the comparison of et7 with et16. The updated p-values and FDRs for *cry2* and *clock* are now reported in the text of the results, since the confidence intervals of the log_2_ FC do not overlap zero (Figure 4), but their FDRs exceed 0.05 (lines 253-256).

7. In the Methods section, the statistical analyses applied are not always clearly described. It is unclear whether the data were normally distributed and whether parametric or non-parametric tests were used. The authors should provide this information.

Response: We have updated and clarified statistical tests throughout the methods.

Minor points
- The authors should provide the raw PRC data in the supplementary materials to improve transparency and reproducibility.

Response: We have added supplementary information (S2 File) with the phase response for each individual and submitted the raw behavior files, phase response, and all analysis code to Zenoda (10.5281/zenodo.21398810) and GitHub (https://github.com/Toporikova-Lab/Spider-RNA-seq-analysis). See also updated Data Availability Statement in the revised manuscript).

- Clarify why only females were used in the study.

Response: For web building species, males leave their web at or just before maturity in search of females. Adult males can thus be extremely difficult to capture in large numbers. For *Metazygia wittfeldae*, we might find 1 male for every 10 females. Given the large numbers of individuals needed for the PRC and gene expression experiments, we chose to limit sampling to females. We have clarified the sex ratio discrepancy in the Methods (lines 417-419).

- The conditions during laboratory acclimation should be described in more detail, including the type and intensity of the light source and the temperature to which the spiders were exposed.

Response: Thank you, we have added this information (lines 419-424).

**Reviewer #2**: This study investigated the mechanisms by which the locomotor activity of the orb weaver, Metazygia wittfeldae, synchronizes with the external light-dark cycle, and the associated gene expression. First, 1-hour light pulses were inserted at various timings, and the resulting changes in behavioral rhythms were observed to obtain phase response curves (PRCs). As a result, type 0 PRCs, suggesting the presence of weak oscillators, were obtained. The PRCs obtained in this study are the first PRCs obtained in spiders. Next, the effects of the presence or absence of light pulses and differences in the timing of light pulse insertion on gene expression were investigated, and some gene groups involved in synchronization with the external cycle were identified.

The manuscript presents a clear line of argument, and the results are interesting. This ms is also written in a way that allows readers who are not experts in chronobiology to fully understand the significance of this research. Although there are no major issues, I provide several comments below.


(Minor comments)
Line 92: “A high amplitude PRC can be indicative of a weak oscillator”
Readers unfamiliar with chronobiology may find it difficult to understand why weak oscillators exhibit high-amplitude PRCs. Adding a brief explanation would make it clearer.

Response: Thank you for the positive response to our manuscript! And thank you for the opportunity to clarify why a high amplitude PRC can be indicative of a weak oscillator. We have added an explanation to the Introduction (lines 92-95).

Literature cited
The journal names of cited papers are sometimes written using their full names and sometimes using abbreviations, resulting in a lack of overall consistency. For example, references 46 and 47 both refer to the same journal (The Journal of Arachnology), but the journal name is written differently. Standardizing the format is desirable.

References 37, 74, 76: Scientific names should be written in italics.

Response: Thank you for pointing out these errors. They have been fixed. Please note that many references were updated and reformatted to comply with journal requirements, but these changes are not highlighted in the track-changes document.

**Reviewer #3**: This manuscript describes the phase response curve (PRC) of a spider species and analyzes changes in gene expression following a light pulse. To my knowledge, this is the first report of a PRC in a spider, and the use of a large number of individuals to consolidate the PRC is commendable. I appreciate the considerable experimental effort invested in this study.
While the construction of the PRC itself appears technically sound, I have major concerns regarding the interpretation of the gene expression data and the conclusions drawn from them.
Due to the limited sampling time points, the gene expression analyses demonstrate only changes in transcript levels following a light pulse, not phase shifts of gene expression rhythms. In addition, although the model adopted by the authors (as indicated in the supplementary information) is based on TIM protein abundance, the manuscript extends this conceptual framework to genome-wide gene expression. This extension is not justified.
As the authors are likely aware, different clock-related and clock-controlled genes exhibit distinct temporal expression profiles: some peak during the photophase, others during the scotophase, and even within the scotophase, peak timing varies substantially among genes. Therefore, interpreting genome-wide expression changes using a model developed for a single clock protein is inappropriate. Consequently, a substantial portion of the Results and Discussion sections is overly speculative and not sufficiently supported by the data.
Overall, I find the PRC analysis itself to be a valuable contribution. However, the subsequent gene expression analyses require substantial reconsideration. The manuscript should strictly focus on conclusions that can be directly supported by the presented data and clearly distinguish empirical results from speculative interpretations.

Response: Thank you for your support of the phase response curve! And yes, thank you for recognizing that we are aware of the variation in expression profiles of core clock genes and clock-controlled genes. We are also aware of the limited interpretations of cycling and phase shifting that can be made from 2 time points. We had thought we acknowledged those limitations in our original manuscript but recognize that the original text could be read as definitive evidence of phase shifting, rather than as suggestive. Furthermore, when reviewing the modified *Neurospora crassa* and *Drosophila melanogaster* models, we came to the same conclusion as the reviewer that it is inappropriate to apply the results of oscillations in regulatory proteins to interpretation of our experimental mRNA abundance. We have thus removed the mathematical models from the manuscript entirely. We have also substantially modified portions of the Results and Discussion regarding the gene expression patterns, which we describe in more detail below.

________________________________________
Specific comments
Introduction
Several definitions require correction or clarification. Daily rhythms observed under light–dark conditions should not be termed “circadian” (L.44–46), as circadian rhythms are defined as rhythms persisting under constant conditions. Likewise, the presence of a rhythm does not directly demonstrate the involvement of a circadian clock (L.47–48). The environmental cue that entrains a rhythm should be referred to as a Zeitgeber (L.69). A more careful and precise explanation of these fundamental concepts is necessary.

Response: Thank you. We have clarified that a daily rhythm is only circadian if it continues in a constant environment, and that these rhythms may be controlled by an internal circadian clock (lines 39-42). We have also expanded the definition of zeitgeber (lines 66-68).

Terminology and notation
L.54, 64. Period → period; Timeless → timeless; Cryptochrome → cryptochrome.
In Drosophila, gene names should not be capitalized.

Response: Thank you for pointing out this error. We have updated throughout.

L.72. CRY1 is introduced without prior explanation.

Response: We have added a sentence introducing cryptochrome paralogs (lines 61-62).

Interpretation
L.113. A wide range of free-running periods does not necessarily indicate a weak oscillator. Please provide appropriate references if this claim is to be retained.

Response: Thank you for the opportunity to clarify. We agree that a wide range of free-running periods does not necessitate a weak oscillator. Our original line of thinking was based on robust entrainment by light even for free-running periods markedly different from 24 hours. We have modified and softened our language accordingly (line 115).

Methods and organization
Section starting at L.129. Because the Materials and Methods section appears later in the manuscript, readers may have difficulty understanding how the PRC experiments were conducted. A brief methodological overview would be helpful, including sex, age, and origin of the spiders; photoperiodic conditions prior to transfer to DD; light pulse conditions; and the activity parameter used to define phase.

Response: Thank you for the opportunity to clarify the underlying experiment for estimating the PRC. We have added a brief overview of the design to the beginning of the Results section (lines 129-137) and expanded quantification of the type 0 classification (lines 154-163).

Figures and Results
L.186. Figures 2C and 2D are referenced before Figures 2A and 2B; the order should be corrected.
L.187–189. The interpretation here is unclear and does not appear to be based on a direct comparison between Figures 2A and 2B. Rather, the data suggest that many genes are downregulated at ET7 compared with ET16 after a light pulse.

Response: Thank you for pointing out the unclear and incorrect reference to panels! We have updated the text discussing Figure 2 (lines 203-209). We should also point out than in revising figures, we chose to reanalyze the differential expression using the current version of R. In the reanalysis, our total counts of down and upregulate genes changed for some comparisons. These numbers have been updated, but do not affect the overall pattern that light tends to cause immediate downregulation of genes.

Figure 2. “log fold change” should be “log2 fold change.”

Response: We have updated throughout.

Gene expression and phase interpretation
As mentioned above, with only two sampling time points, the experiments demonstrate expression changes following a light pulse but do not allow inference of phase shifts in gene expression rhythms. Moreover, as indicated in Figure S2, Figure 3A represents TIM protein abundance, and thus extending this framework to genome-wide expression analyses is not justified. Accordingly, the section from L.214 to L.255 requires major revision or removal.

Response: Thank you for the opportunity to clarify and reframe our experimental gene expression results. We had never intended to use the transcriptome-wide patterns as evidence of regulatory roles in the molecular clock. Instead, our intention had been to use the models as a template for what might happen to mRNA abundance if light shifted the phase of transcript abundance. However, in reviewing the models and our text carefully during the revision process we decided that the models could not be used to support some of our claims. We thus decided to remove the models from the manuscript entirely. For the transcript abundance results, we have limited text in the Results section to be descriptive and have specifically stated that our design is informative about the acute effects of light (lines 189-190).

Discussion
L.347–415. To characterize oscillatory gene expression patterns, substantially denser temporal sampling is required. Chronobiological interpretations based on only two time points are not welcome. The present experiments address the acute effects of a light pulse on gene expression, but do not allow conclusions regarding chronobiological phase regulation.

Response: We agree that 2 time points cannot offer definitive evidence regarding the circadian molecular clock or oscillations in gene expression. However, since our manuscript is one of the only descriptions for spiders of genes known to be involved in the circadian clock, we feel justified in retaining some discussion of the core clock genes. Furthermore, given that 2 other reviewers found the gene expression patterns reported in Figure 3 to be intriguing, we do not wish to completely remove reporting this pattern.

However, we have restricted text in the Results section to be strictly descriptive of patterns and limited potential circadian interpretations to the Discussion section. For the Discussion, we clearly articulate that more sampling times are needed to determine if core clock genes oscillate (lines 364-365, 372-375). We have also modified our discussion of the pattern of flipped expression in response to light between time points to include phase shifting as one option. We now reference the experimental pattern of phase shifting of *per* in *D. melanogaster* instead of the mathematical models (lines 384-387). We also explicitly state that we do not suggest that any of these genes are regulatory, only that they could potentially be outputs of a molecular clock with a light shifted phase (lines 387-390). We also added another potential explanation for the flipped pattern, namely overcompensation of transcription to make up for the initial reduction (lines 390-392). We have otherwise limited the Discussion to the functions of genes that respond to light (lines 393-404).
