# Supplementary material for: Phase response curve and RNA-sequencing demonstrate spiders’ sensitivity to light and pinpoint candidate light-responsive genes
Source: PLoS One. 2026 Sep 23;21(9):e0344063. doi: 10.1371/journal.pone.0344063 (PMC13600489; doi:10.1371/journal.pone.0344063)
Supplement: Peer review history

# Peer review: Decision Letter 0

**DOI:** 10.1371/journal.pone.0344063.r001

**Authors:** Nicholas Foulkes

21 May 2026

Dear Dr. Ayoub,

Please submit your revised manuscript by Jul 05 2026 11:59PM. If you will need more time than this to complete your revisions, please reply to this message or contact the journal office at plosone@plos.org. When you're ready to submit your revision, log on to https://www.editorialmanager.com/pone/ and select the 'Submissions Needing Revision' folder to locate your manuscript file.

- A letter that responds to each point raised by the academic editor and reviewer(s). You should upload this letter as a separate file labeled 'Response to Reviewers'.
- A marked-up copy of your manuscript that highlights changes made to the original version. You should upload this as a separate file labeled 'Revised Manuscript with Track Changes'.
- An unmarked version of your revised paper without tracked changes. You should upload this as a separate file labeled 'Manuscript'.

If you would like to make changes to your financial disclosure, please include your updated statement in your cover letter. Guidelines for resubmitting your figure files are available below the reviewer comments at the end of this letter.

If applicable, we recommend that you deposit your laboratory protocols in protocols.io to enhance the reproducibility of your results. Protocols.io assigns your protocol its own identifier (DOI) so that it can be cited independently in the future. For instructions see: https://journals.plos.org/plosone/s/submission-guidelines#loc-laboratory-protocols. Additionally, PLOS ONE offers an option for publishing peer-reviewed Lab Protocol articles, which describe protocols hosted on protocols.io. Read more information on sharing protocols at https://plos.org/protocols?utm_medium=editorial-email&utm_source=authorletters&utm_campaign=protocols.

As the corresponding author, your ORCID iD is verified in the submission system and will appear in the published article. PLOS supports the use of ORCID, and we encourage all coauthors to register for an ORCID iD and use it as well. Please encourage your coauthors to verify their ORCID iD within the submission system before final acceptance, as unverified ORCID iDs will not appear in the published article. *Only*  the individual author can complete the verification step; PLOS staff *cannot*  verify ORCID iDs on behalf of authors.

We look forward to receiving your revised manuscript.

Kind regards,

Nicholas S. Foulkes, D.Phil

Academic Editor

PLOS One

Journal Requirements:

When submitting your revision, we need you to address these additional requirements.

1. Please ensure that your manuscript meets PLOS ONE's style requirements, including those for file naming. The PLOS ONE style templates can be found at

https://journals.plos.org/plosone/s/file?id=wjVg/PLOSOne_formatting_sample_main_body.pdf and

https://journals.plos.org/plosone/s/file?id=ba62/PLOSOne_formatting_sample_title_authors_affiliations.pdf

2. In your Methods section, please provide additional information regarding the permits you obtained for the work. Please ensure you have included the full name of the authority that approved the field site access and, if no permits were required, a brief statement explaining why.

3. Thank you for stating the following in the Acknowledgments Section of your manuscript:

“We thank Jess Petko for conversations about clock genes. This work was funded by the National Science Foundation (IOS-2235711 to NT and NAA, IOS-2235710 to DM and TCJ).”

We note that you have provided funding information that is not currently declared in your Funding Statement. However, funding information should not appear in the Acknowledgments section or other areas of your manuscript. We will only publish funding information present in the Funding Statement section of the online submission form.

Please remove any funding-related text from the manuscript and let us know how you would like to update your Funding Statement. Currently, your Funding Statement reads as follows:

“This work was supported by funding from the National Science Foundation (NSF): IOS-2235711 to NT and NAA, IOS-2235710 to DM and TCJ. https://www.nsf.gov/ NSF did not play a role in the study design, data collection or analysis, the decision to publish, or preparation of this manuscript.”

Please include your amended statements within your cover letter; we will change the online submission form on your behalf.

4. We note that the grant information you provided in the ‘Funding Information’ and ‘Financial Disclosure’ sections do not match.

When you resubmit, please ensure that you provide the correct grant numbers for the awards you received for your study in the ‘Funding Information’ section.

5. If the reviewer comments include a recommendation to cite specific previously published works, please review and evaluate these publications to determine whether they are relevant and should be cited. There is no requirement to cite these works unless the editor has indicated otherwise.

[Note: HTML markup is below. Please do not edit.]

Reviewers' comments:

Reviewer's Responses to Questions

**Comments to the Author**

1. Is the manuscript technically sound, and do the data support the conclusions?

Reviewer #1: Yes

Reviewer #2: Yes

Reviewer #3: No

**********

2. Has the statistical analysis been performed appropriately and rigorously?

Reviewer #1: Yes

Reviewer #2: Yes

Reviewer #3: Yes

**********

3. Have the authors made all data underlying the findings in their manuscript fully available?

The PLOS Data policy

Reviewer #1: No

Reviewer #2: Yes

Reviewer #3: Yes

**********

4. Is the manuscript presented in an intelligible fashion and written in standard English?

Reviewer #1: Yes

Reviewer #2: Yes

Reviewer #3: Yes

**********

Reviewer #1: This manuscript investigates circadian light entrainment in the orb-weaving spider Metazygia wittfeldae using two complementary approaches: the construction of a phase response curve (PRC) based on 1-hour light pulses provided at different circadian times, and RNA sequencing following a light pulse at CT17 to identify candidate light-responsive genes. The authors report a high-amplitude type 0 PRC characterized by strong phase advances and delays and identify differentially expressed genes at both 1 and 10 hours after the pulse. The integration of behavioural assays and transcriptomic analysis in a non-model arthropod represents a key strength of the study and provides novel data to the field.

The manuscript is generally well written, but I would currently recommend addressing the comments reported below.

1. The authors describe a “type 0 PRC” characterized by high-amplitude phase shifts (>6 h) and a breakpoint at CT17. While this is a potentially important result, further clarification and justification are needed to support this classification. Type 0 PRCs are typically associated with strong resetting stimuli. The manuscript would benefit from more explicit quantitative criteria used to classify the PRC as type 0, as well as a clearer description of whether the transitions between delay and advance phase shifts are sufficiently abrupt to meet canonical definitions.

2. The distinction between type 0 and type 1 PRCs can depend on light intensity. How did you choose the light intensity? Did you test other intensities? The authors used 1,400 lux, stating that it is comparable to sunlight in the early afternoon of an overcast day. However, 1,400 lux is much dimmer than typical daylight, even under overcast conditions. It more likely corresponds to illumination under heavy cloud cover or during early morning or late afternoon conditions.

I understand that many published studies on this topic use lux as a measure of illumination, but this is not ideal because it is based on human visual sensitivity. While it may be acceptable for historical reasons and to allow comparisons across studies, I suggest also reporting irradiance values in physical units (e.g., W/m² or µW/cm²).

3. The authors should clarify key aspects of the experimental design and replication for the behavioural assays. Although more than 100 spiders were used in total, it is important to specify how many individuals were tested for each CT and whether the same individuals were reused across conditions. It should also be clarified whether all measurements are independent.

How consistent were the phase shifts across individuals, given the expected inter-individual variability?

4. What does the abbreviation “et” mean? While ZT is the standard for entrained rhythms, I am not familiar with “et.” Does it refer to “entrainment tme” or “endogenous time”? In addition, the abbreviation “FRP” for free-running period is not commonly used.

5. The observation that genes downregulated one hour after the light pulse become upregulated nine hours later is intriguing. While this pattern is consistent with a phase shift, it does not constitute definitive proof, as it may also reflect transient stress responses or general light-induced pathways.

The authors should avoid language implying mechanistic confirmation and instead frame these findings as the identification of candidate light-responsive genes rather than genes directly underlying entrainment mechanisms.

More broadly, it remains unclear whether the observed gene expression changes reflect a true circadian phase shift or simply acute photic responses. Ideally, sampling across additional time points over a full circadian cycle would help resolve this distinction. If this is not feasible, the limitation should be explicitly acknowledged in the text.

6. In the paragraph describing the differential expression of clock genes, the authors state that some genes are much more highly expressed than others, but no statistical analysis is reported. This information is necessary to support such claims. In addition, it is unclear whether the data shown in the figure represent means. If so, standard deviations (or other measures of variability) are missing. If they are not means, does each value correspond to a single measurement? The figure legend is not clear and should be more explicit.

7. In the Methods section, the statistical analyses applied are not always clearly described. It is unclear whether the data were normally distributed and whether parametric or non-parametric tests were used. The authors should provide this information.

Minor points

- The authors should provide the raw PRC data in the supplementary materials to improve transparency and reproducibility.

- Clarify why only females were used in the study.

- The conditions during laboratory acclimation should be described in more detail, including the type and intensity of the light source and the temperature to which the spiders were exposed.

Reviewer #2: This study investigated the mechanisms by which the locomotor activity of the orb weaver, Metazygia wittfeldae, synchronizes with the external light-dark cycle, and the associated gene expression. First, 1-hour light pulses were inserted at various timings, and the resulting changes in behavioral rhythms were observed to obtain phase response curves (PRCs). As a result, type 0 PRCs, suggesting the presence of weak oscillators, were obtained. The PRCs obtained in this study are the first PRCs obtained in spiders. Next, the effects of the presence or absence of light pulses and differences in the timing of light pulse insertion on gene expression were investigated, and some gene groups involved in synchronization with the external cycle were identified.

The manuscript presents a clear line of argument, and the results are interesting. This ms is also written in a way that allows readers who are not experts in chronobiology to fully understand the significance of this research. Although there are no major issues, I provide several comments below.

(Minor comments)

Line 92: “A high amplitude PRC can be indicative of a weak oscillator”

Readers unfamiliar with chronobiology may find it difficult to understand why weak oscillators exhibit high-amplitude PRCs. Adding a brief explanation would make it clearer.

Literature cited

The journal names of cited papers are sometimes written using their full names and sometimes using abbreviations, resulting in a lack of overall consistency. For example, references 46 and 47 both refer to the same journal (The Journal of Arachnology), but the journal name is written differently. Standardizing the format is desirable.

References 37, 74, 76: Scientific names should be written in italics.

Reviewer #3: This manuscript describes the phase response curve (PRC) of a spider species and analyzes changes in gene expression following a light pulse. To my knowledge, this is the first report of a PRC in a spider, and the use of a large number of individuals to consolidate the PRC is commendable. I appreciate the considerable experimental effort invested in this study.

While the construction of the PRC itself appears technically sound, I have major concerns regarding the interpretation of the gene expression data and the conclusions drawn from them.

Due to the limited sampling time points, the gene expression analyses demonstrate only changes in transcript levels following a light pulse, not phase shifts of gene expression rhythms. In addition, although the model adopted by the authors (as indicated in the supplementary information) is based on TIM protein abundance, the manuscript extends this conceptual framework to genome-wide gene expression. This extension is not justified.

As the authors are likely aware, different clock-related and clock-controlled genes exhibit distinct temporal expression profiles: some peak during the photophase, others during the scotophase, and even within the scotophase, peak timing varies substantially among genes. Therefore, interpreting genome-wide expression changes using a model developed for a single clock protein is inappropriate. Consequently, a substantial portion of the Results and Discussion sections is overly speculative and not sufficiently supported by the data.

Overall, I find the PRC analysis itself to be a valuable contribution. However, the subsequent gene expression analyses require substantial reconsideration. The manuscript should strictly focus on conclusions that can be directly supported by the presented data and clearly distinguish empirical results from speculative interpretations.

________________________________________

Specific comments

Introduction

Several definitions require correction or clarification. Daily rhythms observed under light–dark conditions should not be termed “circadian” (L.44–46), as circadian rhythms are defined as rhythms persisting under constant conditions. Likewise, the presence of a rhythm does not directly demonstrate the involvement of a circadian clock (L.47–48). The environmental cue that entrains a rhythm should be referred to as a Zeitgeber (L.69). A more careful and precise explanation of these fundamental concepts is necessary.

Terminology and notation

L.54, 64. Period → period; Timeless → timeless; Cryptochrome → cryptochrome.

In Drosophila, gene names should not be capitalized.

L.72. CRY1 is introduced without prior explanation.

Interpretation

L.113. A wide range of free-running periods does not necessarily indicate a weak oscillator. Please provide appropriate references if this claim is to be retained.

Methods and organization

Section starting at L.129. Because the Materials and Methods section appears later in the manuscript, readers may have difficulty understanding how the PRC experiments were conducted. A brief methodological overview would be helpful, including sex, age, and origin of the spiders; photoperiodic conditions prior to transfer to DD; light pulse conditions; and the activity parameter used to define phase.

Figures and Results

L.186. Figures 2C and 2D are referenced before Figures 2A and 2B; the order should be corrected.

L.187–189. The interpretation here is unclear and does not appear to be based on a direct comparison between Figures 2A and 2B. Rather, the data suggest that many genes are downregulated at ET7 compared with ET16 after a light pulse.

Figure 2. “log fold change” should be “log2 fold change.”

Gene expression and phase interpretation

As mentioned above, with only two sampling time points, the experiments demonstrate expression changes following a light pulse but do not allow inference of phase shifts in gene expression rhythms. Moreover, as indicated in Figure S2, Figure 3A represents TIM protein abundance, and thus extending this framework to genome-wide expression analyses is not justified. Accordingly, the section from L.214 to L.255 requires major revision or removal.

Discussion

L.347–415. To characterize oscillatory gene expression patterns, substantially denser temporal sampling is required. Chronobiological interpretations based on only two time points are not welcome. The present experiments address the acute effects of a light pulse on gene expression, but do not allow conclusions regarding chronobiological phase regulation.

**********

what does this mean?). If published, this will include your full peer review and any attached files.

If you choose “no”, your identity will remain anonymous but your review may still be made public.

**Do you want your identity to be public for this peer review?**  For information about this choice, including consent withdrawal, please see our Privacy Policy

Reviewer #1: No

Reviewer #2: No

Reviewer #3: No

**********

[NOTE: If reviewer comments were submitted as an attachment file, they will be attached to this email and accessible via the submission site. Please log into your account, locate the manuscript record, and check for the action link "View Attachments". If this link does not appear, there are no attachment files.]

To ensure your figures meet our technical requirements, please review our figure guidelines: https://journals.plos.org/plosone/s/figures

You may also use PLOS’s free figure tool, NAAS, to help you prepare publication quality figures: https://journals.plos.org/plosone/s/figures#loc-tools-for-figure-preparation.

NAAS will assess whether your figures meet our technical requirements by comparing each figure against our figure specifications.

# Author comment: Author response to Decision Letter 1

**DOI:** 10.1371/journal.pone.0344063.r002

26 Jul 2026

Please see detailed response to reviewers in the Response to Reviewers document uploaded with the manuscript files.

Attachment Submitted filename: Response_to_Reviewers.docx

# Editor report: Decision Letter 1

**DOI:** 10.1371/journal.pone.0344063.r003

**Authors:** Nicholas Foulkes

4 Aug 2026

Phase response curve and RNA-sequencing demonstrate spiders' sensitivity to light and pinpoint candidate light-responsive genes

PONE-D-26-08031R1

Dear Dr. Ayoub,

We’re pleased to inform you that your manuscript has been judged scientifically suitable for publication and will be formally accepted for publication once it meets all outstanding technical requirements.

Within one week, you’ll receive an e-mail detailing the required amendments. When these have been addressed, you’ll receive a formal acceptance letter and your manuscript will be scheduled for publication.

An invoice will be generated when your article is formally accepted. Please note, if your institution has a publishing partnership with PLOS and your article meets the relevant criteria, all or part of your publication costs will be covered. Please make sure your user information is up-to-date by logging into Editorial Manager at Editorial Manager® and clicking the ‘Update My Information' link at the top of the page. For questions related to billing, please contact billing support.

If your institution or institutions have a press office, please notify them about your upcoming paper to help maximize its impact. If they’ll be preparing press materials, please inform our press team as soon as possible -- no later than 48 hours after receiving the formal acceptance. Your manuscript will remain under strict press embargo until 2 pm Eastern Time on the date of publication. For more information, please contact onepress@plos.org.

Kind regards,

Nicholas S. Foulkes, D.Phil

Academic Editor

PLOS One

Additional Editor Comments (optional):

Reviewers' comments:

# Editor report: Acceptance letter

**DOI:** 10.1371/journal.pone.0344063.r004

**Authors:** Nicholas Foulkes

PONE-D-26-08031R1

PLOS One

Dear Dr. Ayoub,

I'm pleased to inform you that your manuscript has been deemed suitable for publication in PLOS One. Congratulations! Your manuscript is now being handed over to our production team.

At this stage, our production department will prepare your paper for publication. This includes ensuring the following:

* All references, tables, and figures are properly cited

* All relevant supporting information is included in the manuscript submission,

* There are no issues that prevent the paper from being properly typeset

You will receive further instructions from the production team, including instructions on how to review your proof when it is ready. Please keep in mind that we are working through a large volume of accepted articles, so please give us a few days to review your paper and let you know the next and final steps.

Lastly, if your institution or institutions have a press office, please let them know about your upcoming paper now to help maximize its impact. If they'll be preparing press materials, please inform our press team within the next 48 hours. Your manuscript will remain under strict press embargo until 2 pm Eastern Time on the date of publication. For more information, please contact onepress@plos.org.

You will receive an invoice from PLOS for your publication fee after your manuscript has reached the completed accept phase. If you receive an email requesting payment before acceptance or for any other service, this may be a phishing scheme. Learn how to identify phishing emails and protect your accounts at https://explore.plos.org/phishing.

If we can help with anything else, please email us at customercare@plos.org.

Thank you for submitting your work to PLOS ONE and supporting open access.

Kind regards,

PLOS ONE Editorial Office Staff

on behalf of

Dr. Nicholas S. Foulkes

Academic Editor

PLOS One
